# Role of Fungicide Applications on the Integrated Management of Wheat Stripe Rust

**DOI:** 10.3389/fpls.2020.00733

**Published:** 2020-06-09

**Authors:** Marcelo Carmona, Francisco Sautua, Oscar Pérez-Hérnandez, Erlei M. Reis

**Affiliations:** ^1^Cátedra de Fitopatología, Facultad de Agronomía, Universidad de Buenos Aires, Buenos Aires, Argentina; ^2^School of Agricultural Sciences, Northwest Missouri State University, Maryville, MO, United States; ^3^Escuela Para Graduados “Alberto Soriano”, Facultad de Agronomía, Universidad de Buenos Aires, Buenos Aires, Argentina

**Keywords:** yellow rust, *Puccinia striiformis* f. sp. *tritici (Pst)*, chemical control, integrated disease management, yield loss, new races

## Abstract

First described in Europe in 1777, stripe rust (SR) caused by *Puccinia striiformis* Westend. f. sp. *tritici* Erikss (*Pst*) is one of the most important and destructive diseases of wheat worldwide. Until 2000, SR was mainly endemic to cooler regions, but since then, new aggressive strains have emerged, spread intercontinentally, and caused severe epidemics in warmer regions across the world. This has put SR as a disease that poses a threat to the world food security. At present, the preferred strategy for control of SR is the access to wheat cultivars with adequate levels of SR resistance. However, wheat breeding programs are not sufficiently advanced to cope with the recently emerged *Pst* strains. Under this scenario, foliar fungicide applications have become an important component of SR management, but information on the effects of fungicide applications on SR control and wheat cultivar yield response is scarce. This review seeks to provide an overview of the impact and role of fungicides on SR management. With focus on wheat management in the major wheat-growing regions of the world, the review addresses: (a) the efficacy of different fungicide active ingredients, optimal fungicide timing and number of applications in controlling SR, and (b) the impact of fungicide on wheat grain yield response. Inclusion of fungicides in an integrated crop management approach is discussed.

## Introduction

**Figure 1:**
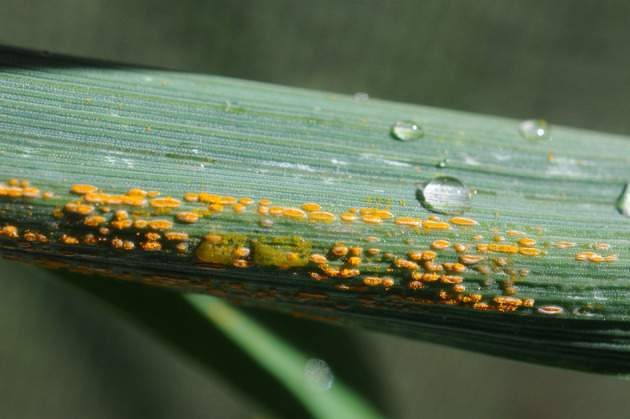
Stripe rust symptoms and signs. The first sign of stripe rust is the appearance of yellow streaks (pre-pustules), followed by small, bright yellow, elongated uredial pustules arranged in conspicuous rows on the leaves. Photo: Dr. Marcelo Carmona, Silvana Di Núbila.

Stripe rust (SR), also called yellow rust, is an old and devastating disease of wheat (*Triticum aestivum* L.) caused by the biotrophic fungus *Puccinia striiformis* f. sp. *tritici* (*Pst*) (Figure 1). Reported in more than 64 countries, SR can severely reduce yield in all wheat-growing regions of the world (Wellings, 2011; Chen et al., 2014; Chen and Kang, 2017a). Beddow et al. (2015) estimated global damages at over 5 million tons of wheat equivalent to a loss of US$979 million per year. In China, average annual yield losses caused by SR have been estimated at 1 million metric tons (Chen, 2005; Chen et al., 2007). In Australia, yield losses caused by SR were estimated at US$200 million per year (Murray and Brennan, 2009), and in the USA at about US$45 million in 1961 (Shaner and Powelson, 1971; Hendrix, 1994). Due to the magnitude of induced losses, SR is now considered the most economically important wheat disease and threat to food security worldwide (Solh et al., 2012; Chaves et al., 2013; Chen et al., 2014; Gangwar et al., 2017; Schwessinger, 2017; Chen, 2020).

The SR can cause significant crop yield reduction in highly susceptible wheat varieties with average losses from 10 to 70% (Chen, 2005, 2014; Chen and Kang, 2017a; Lan et al., 2017; Buendía-Ayala et al., 2019). The pathogen reduces grain yield and quality (Bryson et al., 1997; Chen et al., 2014; He et al., 2019) by: (i) growing, colonizing and reproducing itself at the expense of the energy produced by the plant; (ii) inducing chlorosis and necrosis, which will affect photosynthesis, light interception and light reflectance; (iii) inducing rapid and generalized foliar senescence and poor grain filling; and (iv) reducing root growth (Bever, 1937; Doodson et al., 1964a,b). The magnitude of yield losses will depend on the degree of cultivar resistance to SR and the crop growth stage at the time of epidemic onset. In general, high yield losses are observed in susceptible varieties infected early in the season (Batts and Elliott, 1952; Ash and Brown, 1990; Gaunt and Cole, 1991; Murray et al., 1994, 1995). In Europe, the *Pst* Warrior/Ambition strain severely affected most grown wheat cultivars from 2011 in Europe, with losses exceeding 50% of potential yield (Vergara-Diaz et al., 2015; Hovmøller et al., 2016). In Argentina, wheat yield losses reached a maximum of 4,700 kg ha^−1^ (70%) in fields heavily infected with newly introduced exotic races of *Pst* during the 2017 growing season.

Due to its polycyclic nature, SR epidemics can reach high infection rates in SR susceptible wheat cultivars, especially when temperature and relative humidity are favorable for disease development. Combined, temperature and relative humidity regulate several critical stages of the *Pst* life cycle such us spore germination, infection, latent period, sporulation, spore survival, and host resistance, all of which influence epidemic onset (Rapilly, 1979; Chen et al., 2014; Ma et al., 2015; Grabow et al., 2016). In Luxemburg, a threshold-based weather model for predicting SR infection indicated that temperatures between 4 and 16°C, a minimum of 4 continuous hours of relative humidity >92%, and rainfall ≤ 0.1 mm accurately predicted SR infections (El Jarroudi et al., 2017). In France, de Vallavieille-Pope et al. (1995) observed that SR infection on wheat seedlings required 3.5 h of minimal leaf wetness duration at 12°C.

**Figure 2:**
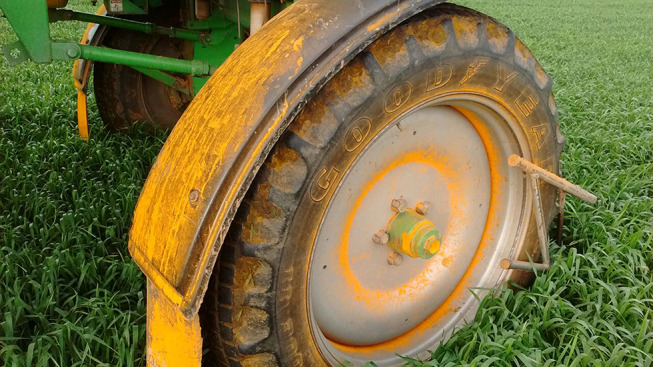
Wheat yellow rust contaminated equipment early in the growing season (September 2017) in Los Cisnes, La Carlota, Cordoba province, Argentina. Photo: Ing. Agr. Juan Pablo Ioele.

**Figure 3:**
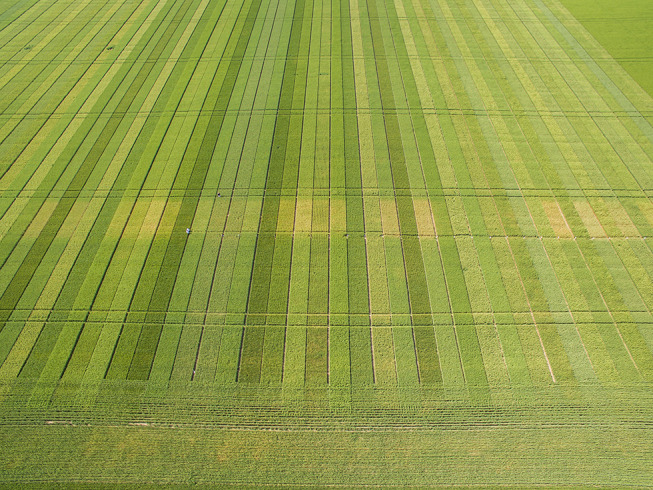
Drone image of stripe rust wheat variety trial. The yellow transverse belt corresponds to different commercial varieties of bread wheat that received no fungicide application. Photo: Ing. Agr. Carlos Grosso, VMV Siembras.

Since the year 2000, new aggressive strains have emerged and spread intercontinentally, causing destructive pandemics in warmer regions across the world (Hovmøller et al., 2008; Loladze et al., 2009; Milus et al., 2009; Wellings, 2011; Mboup et al., 2012; Liu et al., 2017; de Vallavieille-Pope et al., 2018). This new scenario has caused global geographical expansion of SR during the last 20 years and forced the use of fungicides as an essential disease control measure. For instance, in 2017 Argentina faced the worst SR epidemics since the 1930s, with about 3,000,000 affected hectares (Carmona et al., 2019) (Figure 2). The disease spread rapidly throughout most wheat-growing areas of the country. SR was observed on almost all wheat cultivars and developed the typical field infected patches known as “foci” or hot-spots. Depending on the particular crop environment and wheat variety, SR severity ranged from 5 to 50% leaf coverage. The new 2017 and 2018 SR epidemics forced Argentine farmers to perform two applications of fungicide per growing season, on average. Grain yield was negatively correlated with disease severity and field trials showed average yield losses of 3,700 kg ha^−1^ (53%) and up to 4,700 kg ha^−1^ (70%) in severe cases where the disease was not controlled (Figure 3). Economic losses otherwise could be of up to 500 USD ha^−1^ (Carmona et al., 2019).

Although planting SR resistant cultivars is the most efficient and environment friendly method to reduce yield losses due to SR (Line and Chen, 1995; Zhang et al., 2017), 88% of the world's wheat production is based on wheat varieties susceptible to the disease (Beddow et al., 2015). Single race-specific resistance genes (*Yr*) had been effective in protecting wheat crops for several years, thus widely used in breeding programs, until new emerged *Pst* races made them ineffective (Zhang et al., 2017; McIntosh et al., 2018). In this context, while many molecular studies have been undertaken recently to identify genes conferring SR resistance (Ren et al., 2012; Rosewarne et al., 2012; Sharma-Poudyal et al., 2013; Zegeye et al., 2014; Maccaferri et al., 2015; Naruoka et al., 2015; Tang et al., 2015; Cheng et al., 2016; Yang et al., 2016, 2019; Muleta et al., 2017; Wang B. et al., 2017; Wang J. et al., 2017; Klymiuk et al., 2018; Marchal et al., 2018; Nsabiyera et al., 2018; Yuan F. P. et al., 2018; Elbasyoni et al., 2019; Liu et al., 2019; Mu et al., 2019; Rahmatov et al., 2019; Saleem et al., 2019; Wamalwa et al., 2019; Xu et al., 2019; Zeng et al., 2019; Zhang et al., 2019; Ramachandran et al., 2020), the development of commercially available resistant cultivars will still need several years and considerable investment in many countries (Solh et al., 2012; Chaves et al., 2013; Ellis et al., 2014; Beddow et al., 2015). Besides, genetic improvement in search of resistant wheat cultivars is not carried out equally in the different wheat producing regions with different climates.

On the other hand, *Pst* exhibits high degree of genetic variation thus high pathogenic variability. This pathogen possesses the ability to generate several resistance-breaking races or pathotypes that carry different race-specific avirulence or effector coding genes that make them more specialized in infecting different wheat cultivars. *Pst* genomes are highly heterozygous and encode several effectors (Chen et al., 2009, 2010; Cantu et al., 2013; Zheng et al., 2013; Cuomo et al., 2017; Kiran et al., 2017; Schwessinger et al., 2018; Xu et al., 2020). This genetic characteristic results in rapid emergence of virulent *Pst* races. The high variability of this pathogen is due to its high reproducibility, genetic diversity as a result of sexual recombination, long-distance dissemination capacity and ability to adapt to different environments (Jin et al., 2010; Hovmøller et al., 2011, 2016; Jin, 2011; Zhao et al., 2013, 2016; Zheng et al., 2013; Ali et al., 2014a,b,c, 2017; Tian et al., 2016; Wang et al., 2016; Chen and Kang, 2017a; Liu et al., 2017; Wan et al., 2017; Xia et al., 2018; Yuan C. Y. et al., 2018; Schwessinger et al., 2019; Siyoum et al., 2019). Uredioniospores of *Pst* can disperse at continental scales through wind currents and low-level jets (Chen and Kang, 2017a). They can be disseminated by wind from a few meters to more than 8,000 km in the same growing season, or 2,400 km in 6 months (Nagarajan and Singh, 1990; Line and Qayoum, 1992; Hovmøller et al., 2002; Zeng and Luo, 2006). Likewise, urediniospores can also be disseminated from one continent to another in clothing and footwear of long-distance travelers (Wellings et al., 1987).

The rapid inter-continental spread of novel *Pst* aggressive strains emphasizes the global importance of SR. The disease impact is much higher than “normal” when sudden “exotic” incursions of strains from other continents occur. Thus, SR should be considered in many countries of utmost importance by breeders and plant pathologists. Unfortunately, wheat breeding programs are not sufficiently prepared to cope with such strains, which leaves farmers with cultivars highly prone to rusts and with the need to use fungicides. Under the lack of wheat cultivars resistant to all *Pst* races and *Yr* genes are overcome by newly, continuously generated strains (Wan et al., 2016; Singh et al., 2017a,b), SR management relies heavily on the application of fungicides (Kang et al., 2010; Xi et al., 2015; Carmona et al., 2019).

In many regions, the dedication and investment in research on different aspects covering the correct use of fungicides to control cereal rusts (optimal time of application, optimal field dose, effectiveness of new molecules, monitoring sensitivity/resistance to fungicides, etc.) is limited. Despite being crop destructive pathogens, having high epidemiological rates, with genetic variability of races, and some of them with sexual reproduction, many resources for research on cereal rusts have focused on the genetic-molecular identification of races and development of resistant wheat genotypes. Some of the reasons that could explain this scenario could be found in the following two aspects: (i) it is undeniable that genetic resistance has historically achieved a resounding success in the management of several rusts and in particular in the SR management, at least until 2011, when better adapted and more aggressive new races broke the resistance of many cultivars in important wheat regions (Hovmøller et al., 2016). In this way, genetic resistance became the main SR control tool; and (ii) according to Oliver (2014), rusts in general have always been well-controlled with fungicides under field conditions when compared with other pathogens of similar epidemiological characteristics (e.g., *Botrytis, Zymoseptoria*, etc.). That is, the fungicides most used to combat rusts, such as quinone outside inhibitors (QoIs) and demethylation inhibitors (DMIs), have maintained their performance and efficacy either because rusts have an intron that prevents the G143A mutation (which would confer a robust resistance to QoIs); or because DMIs are low-risk resistance molecules that respond to quantitative resistance (Cools et al., 2013; Price et al., 2015). This efficacy maintained over time did not arouse interest in research on the use of fungicides and, therefore, was not a priority. However, an exception deserves to be noted: the case of Asian soybean rust caused by *Phakopsora pachyrhizi*. The availability of genotypes resistant to this particular rust is practically null and the use of fungicides has been massive for several years. Under this scenario, cases of resistance to all kinds of single-site fungicides used (QoIs, DMI, and SDHIs) have already been reported (Schmitz et al., 2014). It is for this reason that for this pathosystem, research in chemical control is a priority. Although in some countries fungicides were always used to control SR, their use has only become more extensive and massive in recent years, coinciding with the emergence of exotic races and the breakdown of genetic resistance. Given this current situation, there is a concern and the need for research on the correct use of fungicides for SR control. In this review, an overview of the impact and role of fungicides on SR management is addressed based on available records worldwide. The efficacy of different fungicide active ingredients, optimal fungicide timing and number of applications in controlling SR and the impact of fungicide on grain yield response are discussed.

## Fungicides Used for Control of SR

Fungicides are chemical compounds that inhibit or eradicate the growth of fungi, fungal-like pathogens, and their spores (Reis and Carmona, 2013). They can be classified conveniently according to at least three important aspects: (1) mobility; (2) mode of action; and (3) time of application in relation to the sub-phases of infection (Hewitt, 1998).

### Classification of Fungicides According to Mobility

#### Non-penetrating Fungicides—Non-Mobile

Non-penetrating fungicides are not absorbed by the plant tissues and therefore not translocated. These fungicides remain on plant surfaces as a protective barrier or “shield” that will inhibit spore germination and/or mycelial growth. Therefore, this type of fungicides can be removed from the plant surface by rain or irrigation. Some examples of non-penetrating fungicides that have been formerly used to control SR include: mancozeb (Gupta et al., 1975), sulfur, maneb (Rathmell and Skidmore, 1982), bordeaux mixture (Liu et al., 1965), cupric salts, nickel salts, etc. (Line, 2002; Chen and Kang, 2017b).

#### Penetrant and Mobile (Systemic) Fungicides

Penetrating fungicides are deposited on plant surfaces, get absorbed by leaf tissues and transported upward by xylem vessels. They may be transported at very short distances (local systemic movement) or over long distances within the plant (true systemic fungicide) from the site of uptake depending on chemical compound attributes.

Mobile fungicides have contributed to improve crop disease control since, unlike non-penetrating fungicides, are not exposed to leaching and photo-decomposition, requiring fewer number of applications. Thus, penetrant-mobile fungicides present high control efficiency in smaller rates per hectare in comparison with non-penetrating fungicides.

Among penetrant-mobile fungicides, those that are more mobile within plant tissues, are transported upward by xylem (acropetal movement). Only a few fungicides can be partially translocated basipetally in the phloem.

The term “mesostemic” was devised to identify a complex group of strobilurins fungicides with diverse characteristics. Mesostemic movement refers to penetrating fungicides that act and move in the mesophyll. Some of them are also translaminar (passes from one leaf face to the other) and systemic (ex. azoxystrobin, picoxystrobin) while others also move through the vapor phase, e.g., trifloxystrobin (Bartlett et al., 2002; Balba, 2007).

### Classification of Fungicides According to Mode of Action

Fungicides interfere with several cellular processes essential to the life of fungi and fungal-like organisms. How a fungicide molecule inhibits the growth or kills a given fungus is refer to its mode or mechanism of action (MOA) and it constitutes one of the way of classification. Currently, Fungicide Resistance Action Committee (FRAC) scheme lists 11 different modes of action (FRAC, 2020).

Different MOAs, applied as seed or foliar treatments, are used to prevent and cure SR. Non-penetrating protective fungicides have been used several years ago in SR control (Line, 2002). Currently, systemic fungicides such us DMI (FRAC Code 3), QoI (FRAC Code 11), and succinate dehydrogenase inhibitors (SDHI) (FRAC Code 7) are the most widely used fungicides worldwide (Chen and Kang, 2017b). According to their chemical structure, within the DMI fungicides the most numerous and important group are the triazoles. Although the fungicides strobilurins and carboxamides have the same MOA (inhibition of mitochondrial respiration), they have different sites of action. Carboxamides act in complex II whereas strobilurins act in complex III (FRAC, 2020). On the other hand, triazoles inhibit ergosterol biosynthesis, an essential component of the fungal cell membrane. Strobilurins, carboxamides and triazoles are single-site fungicides, i.e., they act against a single target or essential enzyme in an important metabolic pathway in a fungus. In contrast, fungicides that inhibit multiple sites, such as maneb, mancozeb, and chlorothalonil, affect several metabolic sites within a fungus.

The most frequent used fungicides to control SR are DMIs: cyproconazole, epoxiconazole, fluquinconazole, flutriafol, metconazole, propiconazole, prothioconazole, and tebuconazole; QoIs: azoxystrobin, kresoxim methyl, trifloxystrobin picoxystrobin, and pyraclostrobin; and SDHIs: fluxapyroxad, bixafen, and benzovindiflupyr (Table 1). Particularly, DMI fungicides have been used extensively to control SR (Chen et al., 1982, 1984; Conner and Kuzyk, 1988; Han et al., 2003, 2006; Chen, 2005; Wan et al., 2007).

**Table 1:** Fungicides currently used for control of stripe rust (SR) in the major wheat-growing regions of the world.

**Country**	**Trade mark**	**Active ingredients (%)**	**Label rate (mL ha^**−1**^)**	**Application rate (grs a.i. ha^**−1**^)**	**Chemical group**	**Mode of action**	**FRAC group**	**Main Action type**	**Plant organ**	**Reference**
Argentina*	Miravis® Triple Pack	Propiconazole (25) + benzovindiflupir (4) + pydiflumetofen (20)	500 + 150 – 600 + 200	125 + 20 + 30 – 150 + 24 + 40	DMI + SDHI + SDHI	C14-demethylase in sterol biosynthesis + succinate-dehydrogenase	7 + 3	Preventive + curative	Foliar	Syngenta Agro S.A.
	Elatus® Ace	Propiconazole (25) + benzovindiflupir (4)	500–600	125 + 20 – 150 + 24	DMI + SDHI	C14-demethylase in sterol biosynthesis + succinate-dehydrogenase	7 + 3	Preventive + curative	Foliar	Syngenta Agro S.A.
	Orquesta Ultra®	Fluxapyroxad (50) + epoxiconazole (50) + pyraclostrobin (81)	1,000–1,200	50 + 50 + 81 – 60 + 60 + 97.2	SDHI + DMI + QoI	Succinate-dehydrogenase + C14-demethylase in sterol biosynthesis + cytochrome bc1 (ubiquinol oxidase)	7 + 3 + 11	Preventive + curative	Foliar	BASF Argentina S.A.
	Allegro®	Kresoxim methyl (12.5) + epoxiconazole (12.5)	750–1,000	93.75 + 93.75 – 125 + 125	QoI + DMI	Cytochrome bc1 (ubiquinol oxidase) + C14-demethylase in sterol biosynthesis	11 + 3	Preventive + curative	Foliar	BASF Argentina S.A.
	Opera®	Pyraclostrobin (13.3) + epoxiconazole (5)	1,000	133 + 50	QoI + DMI	Cytochrome bc1 (ubiquinol oxidase) + C14-demethylase in sterol biosynthesis	11 + 3	Preventive + curative	Foliar	BASF Argentina S.A.
	Sphere max®	Trifloxystrobin (37.5) + cyproconazole (16)	250–400	93.75 + 40 – 150 + 64	QoI + DMI	Cytochrome bc1 (ubiquinol oxidase) + C14-demethylase in sterol biosynthesis	11 + 3	Preventive + curative	Foliar	Bayer Argentina S.A.
	Nanok®	Azoxystrobin (12.5) + flutriafol (12.5)	600–700	75 + 75 – 87.5 + 87.5	QoI + DMI	Cytochrome bc1 (ubiquinol oxidase) + C14-demethylase in sterol biosynthesis	11 + 3	Preventive + curative	Foliar	FMC Argentina
	Duett plus®	Epoxiconazole (3.75) + metconazole (2.75)	1,200	45 + 33	DMI	C14-demethylase in sterol biosynthesis	3	Curative	Foliar	(In registration)
	Amistar xtra®	Azoxystrobin (20) + cyproconazole (8)	400	80 + 32	QoI + DMI	Cytochrome bc1 (ubiquinol oxidase) + C14-demethylase in sterol biosynthesis	11 + 3	Preventive + curative	Foliar	(In registration)
	Cripton®	Trifloxystrobin (15) + prothioconazole (17.5)	700	105 + 122.5	QoI + DMI	Cytochrome bc1 (ubiquinol oxidase) + C14-demethylase in sterol biosynthesis	11 + 3	Preventive + curative	Foliar	(In registration)
	Cripton xpro®	Trifloxystrobin (15) + prothioconazole (17.5) + bixafen (12.5)	700	105 + 122.5 + 87.5	QoI + DMI + SDHI	Cytochrome bc1 (ubiquinol oxidase) + C14-demethylase in sterol biosynthesis + succinate-dehydrogenase	11 + 3 + 7	Preventive + curative	Foliar	(In registration)
	Stinger®	Picoxystrobin (20) + cyproconazole (8)	400	80 + 32	QoI + DMI	Cytochrome bc1 (ubiquinol oxidase) + C14-demethylase in sterol biosynthesis	11 + 3	Preventive + curative	Foliar	(In registration)
	Rubric®	Azoxystrobin (20) + epoxiconazole (10)	500	100 + 50	QoI + DMI	Cytochrome bc1 (ubiquinol oxidase) + C14-demethylase in sterol biosynthesis	11 + 3	Preventive + curative	Foliar	(In registration)
	Azoxy Pro®	Azoxystrobin (20) + cyproconazole (8)	400	80 + 32	QoI + DMI	Cytochrome bc1 (ubiquinol oxidase) + C14-demethylase in sterol biosynthesis	11 + 3	Preventive + curative	Foliar	(In registration)
Australia	Foliarflo C®, Apparent Suntan®, Baytan T®, Triadimenol T®, Proleaf T®	Triadimenol (15) + cypermethrin (0.4)	1.5 L/ton seed	–	DMI	C14-demethylase in sterol biosynthesis	3	Curative	Seed	SARDI, 2020
	Triadimefon 125 EC®, Slingshot®, Triad®, Turret®	Triadimefon (12.5)	500–1,000	63–125	DMI	C14-demethylase in sterol biosynthesis	3	Curative	Foliar	Wanyera et al., 2010
	Aurora®, Prestige®, Slipstream 250 EC®	Propiconazole (25)	500	125	DMI	C14-demethylase in sterol biosynthesis	3	Curative	Foliar	Wanyera et al., 2010
	Orius 430 SC®, Stingray 430®, Tebuconazole 430 SC®	Tebuconazole (43)	290	125	DMI	C14-demethylase in sterol biosynthesis	3	Curative	Foliar	Wanyera et al., 2010
	Accord 125®, Opus 125®	Epoxiconazole (12.5)	500	63	DMI	C14-demethylase in sterol biosynthesis	3	Curative	Foliar	Wanyera et al., 2010
	Jockey® Stayer®, Quantum Pro®	Fluquinconazole (16.7)	3.0 L/ton seed	–	DMI	C14-demethylase in sterol biosynthesis	3	Curative	Seed	SARDI, 2020
	Armour C®, Arrow C®	Flutriafol (10) + cypermethrin (0.4)	100 mL/100 kg seed	–	DMI	C14-demethylase in sterol biosynthesis	3	Curative	Seed	SARDI, 2020
	Impact Endure®, Jubilee®	Flutriafol (50)	100–200	50–100	DMI	C14-demethylase in sterol biosynthesis	3	Curative	Foliar, in-furrow	SARDI, 2020
	Bayonet®	Flutriafol (25)	100–200	25–50	DMI	C14-demethylase in sterol biosynthesis	3	Curative	Foliar, in-furrow	SARDI, 2020
	Opera®	Pyraclostrobin (8.5) + epoxiconazole (6.25)	500–1000	43 + 31 – 85 + 62.5	QoI + DMI	Cytochrome bc1 (ubiquinol oxidase) + C14-demethylase in sterol biosynthesis	11 + 3	Preventive + curative	Foliar	BASF Australia Ltd
	Tilt Xtra 250 EC®	Propiconazole (25) + cyproconazole (8)	250–500	63 + 20 – 125 + 40	DMI	C14-demethylase in sterol biosynthesis	3	Curative	Foliar	Wanyera et al., 2010
	Amistar Xtra®	Azoxystrobin (20) + cyproconazole (8)	400–800	80 + 32 – 160 + 64	QoI + DMI	Cytochrome bc1 (ubiquinol oxidase) + C14-demethylase in sterol biosynthesis	11 + 3	Preventive + curative	Foliar	Wanyera et al., 2010
	Uniform®	azoxystrobin (32.2) + metalaxyl-m (12.4)	200–400	64.4–128.8	QoI	Cytochrome bc1 (ubiquinol oxidase)	11	Preventive	In-furrow	SARDI, 2020
Canada	Quilt®	Azoxystrobin (7.5) + propiconazole (12.5)	1,000	75 + 125	QoI + DMI	Cytochrome bc1 (ubiquinol oxidase) + C14-demethylase in sterol biosynthesis	11 + 3	Preventive + curative	Foliar	Syngenta Canada Inc.
	Trivapro®	Azoxystrobin (10.5) + propiconazole (11.9) + benzovindiflupyr (2.9)	750–1,000	79 + 89 + 22 – 105 + 119 + 29	QoI + DMI + SDHI	Cytochrome bc1 (ubiquinol oxidase) + C14-demethylase in sterol biosynthesis + succinate-dehydrogenase	11 + 3 + 7	Preventive + curative	Foliar	Syngenta Canada Inc.
	Miravis Ace®	Pydiflumetofen (15) + propiconazole (12.5)	1,000	150 + 125	DMI + SDHI	C14-demethylase in sterol biosynthesis + succinate-dehydrogenase	7 + 3	Preventive + curative	Foliar	Syngenta Canada Inc.
	Nufarm Propiconazole®	Propiconazole (41.8)	300	125	DMI	C14-demethylase in sterol biosynthesis	3	Curative	Foliar, in-furrow	Nufarm Agriculture Inc.
	Headline SC®	Pyraclostrobin (25)	300–400	75–100	QoI	Cytochrome bc1 (ubiquinol oxidase)	11	Preventive	Foliar	BASF Canada Inc.
	Prosaro 250 EC®	Prothioconazole (12.5) + tebuconazole (12.5)	800	100 + 100	DMI	C14-demethylase in sterol biosynthesis	3	Curative	Foliar	Bayer CropScience Inc.
	Folicur 250 EW®	Tebuconazole (25)	375–500	94 + 125	DMI	C14-demethylase in sterol biosynthesis	3	Curative	Foliar	Bayer CropScience Inc.
	Hornet^TM^ 432 F	Tebuconazole (43.2)	220–292	95–126	DMI	C14-demethylase in sterol biosynthesis	3	Curative	Foliar	Nufarm Agriculture Inc.
	Delaro 325 SC®	Trifloxystrobin (15) + prothioconazole (17.5)	572	85.8 + 100	QoI + DMI	Cytochrome bc1 (ubiquinol oxidase) + C14-demethylase in sterol biosynthesis	11 + 3	Preventive + curative	Foliar	Bayer CropScience Inc.
	Caramba®	Metconazole (9)	500–700	45–63	DMI	C14-demethylase in sterol biosynthesis	3	Curative	Foliar	BASF Canada Inc.
	Nexicor™	Pyraclostrobin (20) + propiconazole (12.5) + fluxapyroxad (3)	500	100 + 63 + 45	QoI + DMI + SDHI	Cytochrome bc1 (ubiquinol oxidase) + C14-demethylase in sterol biosynthesis + succinate-dehydrogenase	11 + 3 + 7	Preventive + curative	Foliar	BASF Canada Inc.
	Twinline™	Pyraclostrobin (13) + metconazole (8)	380–500	49 + 30 – 65 + 40	QoI + DMI	Cytochrome bc1 (ubiquinol oxidase) + C14-demethylase in sterol biosynthesis	11 + 3	Preventive + curative	Foliar	BASF Canada Inc.
	Stratego 250 EC®	Trifloxystrobin (12.5) + prothioconazole (12.5)	500	63 + 63	QoI + DMI	Cytochrome bc1 (ubiquinol oxidase) + C14-demethylase in sterol biosynthesis	11 + 3	Preventive + curative	Foliar	Bayer CropScience Inc.
China	Bayleton®	Triadimenol (12.5)	500	63	DMI	C14-demethylase in sterol biosynthesis	3	Curative	Foliar	Wan et al., 2007
	Tilt®	Propiconazole			DMI	C14-demethylase in sterol biosynthesis	3	Curative	Foliar	Chen, 2007
	Stratego®	Propiconazole + trifloxystrobin			DMI + QoI	C14-demethylase in sterol biosynthesis + cytochrome bc1 (ubiquinol oxidase)	3 + 11	Curative + preventive	Foliar	Chen, 2007
	Prosaro®	Prothioconazole + tebuconazole			DMI	C14-demethylase in sterol biosynthesis	3	Curative	Foliar	Chen, 2007
	Quilt®	Propiconazole + azoxystrobin			DMI + QoI	C14-demethylase in sterol biosynthesis + cytochrome bc1 (ubiquinol oxidase)	3 + 11	Curative + preventive	Foliar	Chen, 2007
	Evito®	Fluoxastrobin			QoI	Cytochrome bc1 (ubiquinol oxidase)	11	Preventive	Foliar	Chen, 2007
	Quadris®	Azoxystrobin			QoI	Cytochrome bc1 (ubiquinol oxidase)	11	Preventive	Foliar	Chen, 2007
Egypt	Tilt 250 EC®	Propiconazole (25)	500	125	DMI	C14-demethylase in sterol biosynthesis	3	Curative	Foliar	Wanyera et al., 2010
Europe	Opus Team®	Epoxiconazole (8.4) + fenpropimorph (25)	1,500	126	DMI	C14-demethylase in sterol biosynthesis	3	Curative	Foliar	Jørgensen et al., 2018
	Proline 250 EC®	Prothioconazole (25)	800	200	DMI	C14-demethylase in sterol biosynthesis	3	Curative	Foliar	Jørgensen et al., 2018
	Caramba 90®	Metconazole (9)	1,000	90	DMI	C14-demethylase in sterol biosynthesis	3	Curative	Foliar	Jørgensen et al., 2018
	Folicur 250 EW®	Tebuconazole (25)	1,000	250	DMI	C14-demethylase in sterol biosynthesis	3	Curative	Foliar	Jørgensen et al., 2018
	Osiris®	Epoxiconazole (5.625) + metconazole (4.125)	2,000	112.5 + 82.5	DMI	C14-demethylase in sterol biosynthesis	3	Curative	Foliar	Jørgensen et al., 2018
	Prosaro 250 EC®	Tebuconazole (12.5) + prothioconazole (12.5)	1,000	125	DMI	C14-demethylase in sterol biosynthesis	3	Curative	Foliar	Jørgensen et al., 2018
India	Nativo 75 WG®	Trifloxystrobin (25) + tebuconazole (50)	300–400	75 + 150 – 100 + 200	QoI + DMI	Cytochrome bc1 (ubiquinol oxidase) + C14-demethylase in sterol biosynthesis	11 + 3	Preventive + curative	Foliar	Singh et al., 2016
	Opera®	Pyraclostrobin (13.3) + epoxiconazole (5)	1,000	133 + 50	QoI + DMI	Cytochrome bc1 (ubiquinol oxidase) + C14-demethylase in sterol biosynthesis	11 + 3	Preventive + curative	Foliar	Singh et al., 2016
	Amistar 250 SC®	Azoxystrobin (25)								Singh et al., 2016
	Score 250 EC®	Difenoconazole (25)	500	125	DMI	C14-demethylase in sterol biosynthesis	3	Curative	Foliar	Singh et al., 2016
	Tilt 250 EC®	Propiconazole (25)								Singh et al., 2016
	Folicur 250 EC®	Tebuconazole (25)								Singh et al., 2016
	Bayleton 50 WP®	Triadimefon (50)	500–1,000	250–500						Singh et al., 2016
	Topas 100 EC®	Penconazole (10)								Singh et al., 2016
	Dithane 75 WP®	Mancozeb (7.5)								Singh et al., 2016
	Predict®	Azoxystrobin (11) + tebuconazole (18.3)	741 (gr)	82 + 136	QoI + DMI	Cytochrome bc1 (ubiquinol oxidase) + C14-demethylase in sterol biosynthesis	11 + 3	Preventive + curative	Foliar	
Kenya	Tilt 250 EC®	Propiconazole (25)	500	125	DMI	C14-demethylase in sterol biosynthesis	3	Curative	Foliar	Wanyera et al., 2010
	Nativo 300SC®	Trifloxystrobin (10) + tebuconazole (20)	1,250	125 + 250	QoI + DMI	Cytochrome bc1 (ubiquinol oxidase) + C14-demethylase in sterol biosynthesis	11 + 3	Preventive + curative	Foliar	Wanyera et al., 2016
	Amistar Xtra®	Azoxystrobin (20) + cyproconazole (8)	400	80 + 32	QoI + DMI	Cytochrome bc1 (ubiquinol oxidase) + C14-demethylase in sterol biosynthesis	11 + 3	Preventive + curative	Foliar	Syngenta Inc.
	Cherokee 487.5 SE®	Chlorothalonil (37.5) + propiconazole (6.25) + cyproconazole (5)	1,200	450 + 75 + 60	M + DMI	Multisite + C14-demethylase in sterol biosynthesis	M05 + 3	Preventive + curative	Foliar	Syngenta Inc.
New Zealand	Alto 100 SL®	Cyproconazole (10)	400	40	DMI	C14-demethylase in sterol biosynthesis	3	Curative	Foliar	Wanyera et al., 2010
	Tilt EC®	Propiconazole (25)	500	125	DMI	C14-demethylase in sterol biosynthesis	3	Curative	Foliar	Wanyera et al., 2010
	Opus 125®	Epoxiconazole (12.5)	500	63	DMI	C14-demethylase in sterol biosynthesis	3	Curative	Foliar	Wanyera et al., 2010
	Systiva®	Fluxapyroxad (6.25)	0.75–1.25 lt per ton of seed		SDHI	Succinate-dehydrogenase	7	Preventive	Seed	Beresford, 2011
Pakistan	Tilt®	Propiconazole (41.8)	292	122.1	DMI	C14-demethylase in sterol biosynthesis	3	Curative	Foliar	Syngenta Inc.
Russia	Alto 100 SL®	Cyproconazole (10)	400	40	DMI	C14-demethylase in sterol biosynthesis	3	Curative	Foliar	Syngenta Inc.
South Africa	Abacus® Advance	Epoxiconazole (6.25) + pyraclostrobin (6.25)	1,000	62.5 + 62.5	DMI + QoI	C14-demethylase in sterol biosynthesis + cytochrome bc1 (ubiquinol oxidase)	3 + 11	Curative + preventive	Foliar	ARC Small Grain Institute, 2014
	Acanto 250 SC®	Picoxystrobin (25)	300	75	QoI	Cytochrome bc1 (ubiquinol oxidase)	11	Preventive	Foliar	ARC Small Grain Institute, 2014
	Amistar Xtra®	Azoxystrobin (20) + cyproconazole (8)	500	100 + 40	QoI + DMI	Cytochrome bc1 (ubiquinol oxidase) + C14-demethylase in sterol biosynthesis	11 + 3	Preventive + curative	Foliar	Wanyera et al., 2010
	Folicur 250 EW®	Tebuconazole (25)	750	187.5	DMI	C14-demethylase in sterol biosynthesis	3	Curative	Foliar	ARC Small Grain Institute, 2014
	Bumper 250 EC®, Tilt®	Propiconazole (25)	400	100	DMI	C14-demethylase in sterol biosynthesis	3	Curative	Foliar	Wanyera et al., 2010
	Acanto 250 SC + Capitan 250 EW®	Picoxystrobin (25) + flusilazole (25)	300 + 400	75 + 100	QoI + DMI	Cytochrome bc1 (ubiquinol oxidase) + C14-demethylase in sterol biosynthesis	11 + 3	Preventive + curative	Foliar	ARC Small Grain Institute, 2014
	Cabrio/Folicur	Pyraclostrobin (25)/tebuconazole (25)	250/750	62.5 + 187.5	QoI + DMI	Cytochrome bc1 (ubiquinol oxidase) + C14-demethylase in sterol biosynthesis	11 + 3	Preventive + curative	Foliar	ARC Small Grain Institute, 2014
	Prosper Trio/Prosaro 250 EW®	Tebuconazole (16.7) + triadimenol (4.3) + spiroxamine (25)/prothioconazole (12.5) + tebuconazole (12.5)	500/400	83.5 + 21.5 + 125/50 + 50	QoI + DMI + morpholine	Cytochrome bc1 (ubiquinol oxidase) + C14-demethylase/14-reductase and 8–7 isomerase in sterol biosynthesis	11 + 3 + 5	Preventive + curative	Foliar	ARC Small Grain Institute, 2014
UK	Proline®	Prothioconazole (27.5)	720	198	DMI	C14-demethylase in sterol biosynthesis	3	Curative	Foliar	AHDB, 2019
	Bassoon®	Epoxiconazole (8.3)	1,500	124.5	DMI	C14-demethylase in sterol biosynthesis	3	Curative	Foliar	AHDB, 2019
	Imtrex®	Fluxapyroxad (6.25)	2,000	125	SDHI	Succinate-dehydrogenase	7	Preventive	Foliar	AHDB, 2019
	Comet®	Pyraclostrobin (20)	1,250	250	QoI	Cytochrome bc1 (ubiquinol oxidase)	11	Preventive	Foliar	AHDB, 2019
	Amistar®	Azoxystrobin (25)	1,000	250	QoI	Cytochrome bc1 (ubiquinol oxidase)	11	Preventive	Foliar	AHDB, 2019
	Amistar Opti®	Azoxystrobin (10) + chlorothalonil (50)	1,500	150 + 750	QoI + M	Cytochrome bc1 (ubiquinol oxidase) + Multisite	11 + M05	Preventive	Foliar	AHDB, 2019
	Mobius®	Trifloxystrobin (15) + prothioconazole (17.5)	1,000	150 + 175	QoI + DMI	Cytochrome bc1 (ubiquinol oxidase) + C14-demethylase in sterol biosynthesis	11 + 3	Preventive + curative	Foliar	AHDB, 2019
	Ascra Xpro®	Bixafen (6.5) + fluopyram (6.5) + prothioconazole (13)	1,500	97.5 + 97.5 + 195	SDHI + SDHI + DMI	Succinate-dehydrogenase + C14-demethylase in sterol biosynthesis	7 + 3	Preventive + curative	Foliar	AHDB, 2019
	Elatus Era®	Benzovindiflupyr (7.5) + prothioconazole (15)	1,000	75 + 150	SDHI + DMI	Succinate-dehydrogenase + C14-demethylase in sterol biosynthesis	7 + 3	Preventive + curative	Foliar	AHDB, 2019
	Revystar XE®	Fluxapyroxad (4.75) + mefentrifluconazole (10)	1,500	150 + 71.25	SDHI + DMI	Succinate-dehydrogenase + C14-demethylase in sterol biosynthesis	7 + 3	Preventive + curative	Foliar	AHDB, 2019
Uruguay*	StigmarXtra®	Azoxistrobin (25) + ciproconazol (10)	350	87.5 + 35	QoI + DMI	Cytochrome bc1 (ubiquinol oxidase) + C14-demethylase in sterol biosynthesis	11 + 3	Preventive + curative	Foliar	Germán et al., 2018
	Opera®	Pyraclostrobin (13.3) + epoxiconazole (5)	1,000	133 + 50	QoI + DMI	Cytochrome bc1 (ubiquinol oxidase) + C14-demethylase in sterol biosynthesis	11 + 3	Preventive + curative	Foliar	Germán et al., 2018
	Stratego®	Trifloxystrobin (12.5) + propiconazole (12.5)	500–750	62.5 + 62.5 – 93.75 – 93.75	QoI + DMI	Cytochrome bc1 (ubiquinol oxidase) + C14-demethylase in sterol biosynthesis	11 + 3	Preventive + curative	Foliar	Germán et al., 2018
	Allegro®	Kresoxim methyl (12.5) + epoxiconazole (12.5)	1,000	125 + 125	QoI + DMI	Cytochrome bc1 (ubiquinol oxidase) + C14-demethylase in sterol biosynthesis	11 + 3	Preventive + curative	Foliar	Germán et al., 2018
	Abacus SC	Pyraclostrobin (26) + epoxiconazole (16)	500	130 + 80	QoI + DMI	Cytochrome bc1 (ubiquinol oxidase) + C14-demethylase in sterol biosynthesis	11 + 3	Preventive + curative	Foliar	Germán et al., 2018
	Stigmar Plus®	Azoxistrobin (20) + tebuconazole (15)	500	100 + 75	QoI + DMI	Cytochrome bc1 (ubiquinol oxidase) + C14-demethylase in sterol biosynthesis	11 + 3	Preventive + curative	Foliar	Germán et al., 2018
	Xantho®	Pyraclostrobin (8.1) + epoxiconazole (5) + fluxapyroxad (5)	1,200	97.2 + 60 + 60	QoI + DMI + SDHI	Cytochrome bc1 (ubiquinol oxidase) + C14-demethylase in sterol biosynthesis + succinate-dehydrogenase	11 + 3 + 7	Preventive + curative	Foliar	Germán et al., 2018
USA	Aproach SC®	Picoxystrobin (22.5)	439–877	98.8–197.3	QoI	Cytochrome bc1 (ubiquinol oxidase)	11	Preventive	Foliar	Wegulo, 2015
	Tilt 3.6 EC®, Bumper 41.8 EC®, Fitness®, PropiMax 3.6 EC®	Propiconazole (41.8)	292	122.1	DMI	C14-demethylase in sterol biosynthesis	3	Curative	Foliar	Wegulo, 2015; Chen and Kang, 2017b
	Proline 480 SC®	Prothioconazole (41)	366–417	150–171	DMI	C14-demethylase in sterol biosynthesis	3	Curative	Foliar	Wegulo, 2015
	Folicur 3.6 F®, Embrace®, Monsoon®, Muscle 3.6 F®, Onset 3.6 L®, Tebucon 3.6 F®, Tebustar 3.6 F®, Tebuzol 3.6 F®, Tegrol®, Toledo 3.6 F®	Tebuconazole (38.7)	292	113	DMI	C14-demethylase in sterol biosynthesis	3	Curative	Foliar	Wegulo, 2015; Chen and Kang, 2017b
	Prosaro 421 SC®	Prothioconazole (19) + tebuconazole (19)	475–599	90.3 – 113.8 + 90.3 – 113.8	DMI	C14-demethylase in sterol biosynthesis	3	Curative	Foliar	Chen and Kang, 2017b
	Quilt 200 SC®	Azoxystrobin (7) + propiconazole (11.7)	768–1,023	54 + 90 – 72 + 120	QoI + DMI	Cytochrome bc1 (ubiquinol oxidase) + C14-demethylase in sterol biosynthesis	11 + 3	Preventive + curative	Foliar	Wegulo, 2015
	Quilt Xcel 2.2 SE®, Avaris 2XS®	Azoxystrobin (13.5) + propiconazole (11.7)	768–1,023	103.7 – 138.1 + 90 – 119.7	QoI + DMI	Cytochrome bc1 (ubiquinol oxidase) + C14-demethylase in sterol biosynthesis	11 + 3	Preventive + curative	Foliar	Wegulo, 2015
	Miravis Ace SE®	Pydiflumetofen (13.7) + propiconazole (11.4)	1,000	137 + 114	SDHI + DMI	Succinate-dehydrogenase + C14-demethylase in sterol biosynthesis	7 + 3	Preventive + curative	Foliar	NCERA, 2019
	Stratego 250 EC®	Trifloxystrobin (11.4) + prothioconazole (11.4)	731	83.3 + 83.3	QoI + DMI	Cytochrome bc1 (ubiquinol oxidase) + C14-demethylase in sterol biosynthesis	11 + 3	Preventive + curative	Foliar	Chen and Kang, 2017b
	Stratego YLD®	Trifloxystrobin (32.3) + prothioconazole (10.8)	292	94.3 + 31.5	QoI + DMI	Cytochrome bc1 (ubiquinol oxidase) + C14-demethylase in sterol biosynthesis	11 + 3	Preventive + curative	Foliar	Wegulo, 2015
	Aproach Prima SC®	Picoxystrobin (17.94) + cyproconazole (7.17)	249–497	44.7 – 89.2 + 17.9 – 35.6	QoI + DMI	Cytochrome bc1 (ubiquinol oxidase) + C14-demethylase in sterol biosynthesis	11 + 3	Preventive + curative	Foliar	Wegulo, 2015
	Topguard®	Flutriafol (11.8)	366–512	43–60	DMI	C14-demethylase in sterol biosynthesis	3	Curative	Foliar	WSU, 2019
	Topguard EQ®	Azoxystrobin (25.3) + flutriafol (18.63)	219–292	55 + 41 – 74 + 54	QoI + DMI	Cytochrome bc1 (ubiquinol oxidase) + C14-demethylase in sterol biosynthesis	11 + 3	Preventive + curative	Foliar	WSU, 2019
	Lucento®	Bixafen (15.55) + flutriafol (26.47)	219–402	34 + 58 – 63 + 106	SDHI + DMI	Succinate-dehydrogenase + C14-demethylase in sterol biosynthesis	7 + 3	Preventive + curative	Foliar	WSU, 2019
	Alto + NIS 0.25% v/v®	Cyproconazole (8.9)	402	36	DMI	C14-demethylase in sterol biosynthesis	3	Curative	Foliar	WSU, 2019
	Prosaro 421 SC + NIS 0.125% v/v ®	Prothioconazole (19) + tebuconazole (19)	366	19 + 19	DMI	C14-demethylase in sterol biosynthesis	3	Curative	Foliar	WSU, 2019
	Trivapro + NIS 0.25% v/v®	Azoxystrobin (10.5) + propiconazole (11.9) + benzovindiflupyr (2.9)	687–1,000	72 + 82 + 20 – 105 + 119 + 29	QoI + DMI + SDHI	Cytochrome bc1 (ubiquinol oxidase) + C14-demethylase in sterol biosynthesis + succinate-dehydrogenase	11 + 3 + 7	Preventive + curative	Foliar	WSU, 2019
	Custodia^TM^	Azoxystrobin (11) + tebuconazole (18.3)	750	82.5 + 137.3	QoI + DMI	Cytochrome bc1 (ubiquinol oxidase) + C14-demethylase in sterol biosynthesis	11 + 3	Preventive + curative	Foliar	Chen and Kang, 2017b
	DexterTM Max XCEL®	Azoxystrobin (3.09) + tebuconazole (2.72) + mancozeb (27.25)	3,500	108 + 95 + 956	QoI + DMI + M	Cytochrome bc1 (ubiquinol oxidase) + C14-demethylase in sterol biosynthesis + Multisite	11 + 3 + M03	Preventive + curative	Foliar	WSU, 2019
	Evito 480 SC®	Fluoxastrobin (40.3)	146–292	58.9–117.8	QoI	Cytochrome bc1 (ubiquinol oxidase)	11	Preventive	Foliar	WSU, 2018
	Headline SC®	Pyraclostrobin (22.5)	439–658	98.8–148.1	QoI	Cytochrome bc1 (ubiquinol oxidase)	11	Preventive	Foliar	WSU, 2018
	Caramba 0.75 SL®	Metconazole (8.6)	731–1,243	62.9–106.9	DMI	C14-demethylase in sterol biosynthesis	3	Curative	Foliar	WSU, 2018
	TwinLine 1.75 EC®	Pyraclostrobin (12) + metconazole (7.4)	512–658	61.4 – 79 + 37.9 – 48.7	QoI + DMI	Cytochrome bc1 (ubiquinol oxidase) + C14-demethylase in sterol biosynthesis	11 + 3	Preventive + curative	Foliar	WSU, 2018
	Priaxor®	Pyraclostrobin (28.6) + fluxapyroxad (14.3)	292–585	83.5 – 167.3 + 41.8 – 83.7	QoI + SDHI	Cytochrome bc1 (ubiquinol oxidase) + succinate-dehydrogenase	11 + 7	Preventive	Foliar	WSU, 2018
	Absolute Maxx SC®	Trifloxystrobin (22.6) + tebuconazole (22.6)	366	82.7 + 82.7	QoI + DMI	Cytochrome bc1 (ubiquinol oxidase) + C14-demethylase in sterol biosynthesis	11 + 3	Preventive + curative	Foliar	WSU, 2018
	Delaro 325 SC®	Trifloxystrobin (13.7) + prothioconazole (16)	585	80.1 + 93.6	QoI + DMI	Cytochrome bc1 (ubiquinol oxidase) + C14-demethylase in sterol biosynthesis	11 + 3	Preventive + curative	Foliar	WSU, 2018
	Nexicor EC®	Pyraclostrobin (18.7) + propiconazole (11.7) + fluapyroxad (2.8)	512–950	96 + 60 + 14 – 178 + 111 + 27	QoI + DMI + SDHI	Cytochrome bc1 (ubiquinol oxidase) + C14-demethylase in sterol biosynthesis + succinate-dehydrogenase	11 + 3 + 7	Preventive + curative	Foliar	WSU, 2018
	Preemptor SC®	Fluoxastrobin (14.8) + flutriafol (19.3)	292–439	43 + 56 – 65 + 85	QoI + DMI	Cytochrome bc1 (ubiquinol oxidase) + C14-demethylase in sterol biosynthesis	11 + 3	Preventive + curative	Foliar	WSU, 2018
	Vertisan®	Penthiopyrad (20.6)	731–1,754	151–361.3	SDHI	Succinate-dehydrogenase	7	Preventive	Foliar	Chen and Kang, 2017b
	Viathon®	Tebuconazole (3.3) + potassium phosphite (49)	2,340	70 + 1147	DMI +	C14-demethylase in sterol biosynthesis + host plant defense induction	3 + P 07 (33)	Curative + preventive	Foliar	Chen and Kang, 2017b
	Quadris®, Equation 2.08 SC®	Azoxystrobin (22.9)	292–877	67–200	QoI	Cytochrome bc1 (ubiquinol oxidase)	11	Preventive	Foliar	French, 2016

* *Currently in the registration process*.

### Classification of Fungicides According to Sub-phases of the Affected Infectious Process

In this classification scheme, fungicides are grouped according to which subphase or event of the pathogenesis is affected (Reis and Carmona, 2013). Pathogenesis or disease cycle is a series of dynamic events that occur in succession during a parasitic relationship of a pathogen and a host that leads to development and establishment of infection. The complete disease cycle includes spore dispersal and deposition, spore germination and penetration, infection, host colonization and invasion, reproduction, dissemination, and survival (Agrios, 2005). The incubation period is the time from the beginning of infection till the appearance of first symptoms. Latent period is the time from the beginning of infection until the appearance of first signs. Based on which sub-phase of the infectious process is affected, fungicides can be classified as preventive, curative, and eradicant (Hewitt, 1998).

#### Protectant or Preventive Fungicide

Preventive or protectant fungicides act before fungal spores are deposited or before spore germination occurs. The main action exerted by the fungicide is “protectant” or “pre-penetration.” The fungicide prevents penetration and infection. All non-penetrating fungicides should be considered preventive o protectant agents. Some penetrating fungicides (strobilurins and carboxamides) can also have preventive or protective action (Bartlett et al., 2002; Amaro et al., 2019). This is explained by their mechanism of action based on the inhibition of mitochondrial respiration, a process that is critical during germination of spores.

#### Curative—Penetrating Fungicide

In this case, the fungicide is able to inhibit fungal growth inside the plant tissues before symptoms and signs are observed. They act mainly during the incubation period, paralyzing the infectious process. Disease control occurs after infection but without the presence of symptoms. Triazoles are typically curative fungicides and are frequently used in the control of rusts.

#### Eradicant Fungicides—Penetrating Fungicide

The eradication activity of a fungicide is related to the inhibition of disease progress after the appearance of symptoms or signs. Complete eradication of the pathogenic fungus within the host tissues is rare and difficult to achieve in the field (Ivic, 2010). Most fungicides do not have a significant eradication action, being their preventive and/or curative activities the main attributes to achieve efficient controls.

### Fungicide Seed Treatments

Systemic fungicides applied on seeds are up-taken by seed tissues and seminal roots during seed germination and seedling establishment and then mobilized through the xylem to the plumule and seedling leaves (Reis and Carmona, 2013). Thus, certain systemic fungicides are used as seed treatments to protect seedling leaves exposed to early deposition of SR spores.

Fungicide seed treatments for SR control is beneficial especially in regions where highly susceptible varieties are grown, the disease is frequent, and *Pst* attacks occurs at early wheat vegetative stages. Sometimes, fields that were planted to seed efficiently treated against SR can delay or decrease the number of foliar applications (Rakotondradona and Line, 1984; Brown et al., 1985; Ahanger et al., 2014; Chen and Kang, 2017b; Hollaway, 2019). The major problem with fungicide seed treatment is the plausible phytotoxic effects on the plant stand, especially when high doses of a.i. are used (Rakotondradona and Line, 1984).

The first research on fungicide uptake in seed or soil for SR control were carried out by Powelson and Shaner (1966) and Hardison (1966, 1975a,b). The molecules evaluated were oxathiin penetrant-mobile fungicides: carboxin, oxicarboxin and several substituted analogs of carboxin. Subsequently, other molecules, such as butrizol, triadimenol, triadimefon, were evaluated (Rakotondradona and Line, 1984; Chan, 1985; Scott and Line, 1985; Cheer et al., 1990; Chen and Kang, 2017b). Triadimefon has shown high efficacy in controlling SR and has been one of the most used fungicides in China (Wan et al., 2007). Another DMI fungicide, such as fenpropimorph (morpholine, FRAC Code 5) was also effective for SR control (Conner and Kuzyk, 1988).

Currently, some triazoles such us triticonazole, flutriafol, fluquinconazole and the new carboxamide fluxapiroxad that showed high control efficiency are widely recommended as a seed treatment for SR control (Boshoff et al., 2003; APVMA, 2015; Hollaway, 2019; Wallwork and Garrard, 2020). Seed treatment for SR control should be considered as part of the integrated disease program.

### Foliar Fungicide Application

The first fungicides that provided effective SR control were non-penetrating and protective. However, their use was limited due to the need to be applied repeatedly and because of their ineffectiveness on established infections (Line, 2002). Other fungicides, such as nickel salts (Hardison, 1963) and even antibiotics, such as phleomycin (Purdy, 1964) gave satisfactory SR control. A great advance in the development of fungicides for the control of SR was the discovery of oxathiin fungicides, especially oxycarboxin, which significantly improved SR control (Line, 2002). However, in field trials conducted in Canada, oxycarboxin was not effective for the control of SR (Conner and Kuzyk, 1988).

Subsequently, the development of new systemic molecules increased the possibilities of chemical control. In the USA, since the 1970s numerous tests have been carried out to assess different fungicide molecules and their control efficacy against SR (Line, 2002; Chen and Kang, 2017b). A milestone in the history of SR chemical control was the devastating epidemics of both leaf and stripe rust that occurred in the U.S. Pacific Northwest in 1980 and 1981. According to Line (1993, 2002), the epidemic forced the registration of the fungicide triadimefon, which had proved to be the most efficient molecule in annual field trials. Use of this fungicide reduced and avoided large economic losses due to SR. Similarly, faced with the emergence of unexpected SR epidemics caused by new exotic *Pst* races, it also took time to register fungicides for SR control in several countries, such as Canada. Between 1981 and 1986, triadimefon was made available in Alberta to control SR but on a temporary basis. Afterwards, propiconazole was given restricted registration for SR control (Conner and Kuzyk, 1988).

The search for new and efficient fungicidal molecules and the assessment of their greenhouse and field efficacies continued incessantly. Since the 1980s, DMIs began to be protagonists not only for the control of SR but also for other foliar fungal diseases. Strobilurins were introduced at the end of the 1990s and were widely disseminated in most crops. Although numerous cases of fungicide resistance to this chemical family have emerged (FRAC, 2020), they still maintain efficiency against rusts (Jørgensen et al., 2018). One exception is the Asian soybean rust, for which sensitivity reduction has been reported (Schmitz et al., 2014). Since ~2010, the SDHIs have been introduced in all fungicide markets in the world. This fungicide group successfully complemented QoIs and DMIs to control the complex of fungal diseases that infect wheat. Nevertheless, they are classified by FRAC as high risk fungicides.

Currently, there is a great diversity of fungicide commercial formulations containing a single or several active ingredients in mixture that are registered and/or recommended for SR control in all wheat-growing regions in the world (Table 1). Although the fungicide active ingredients may differ in their effectiveness according to the field dose (Sharma et al., 2016), most of the registered fungicides have shown high SR control when properly applied. The QoI + DMI mixture or mixtures with SDHIs are excellent options because in addition to controlling SR they extend control to other wheat diseases.

For example, in Argentina, the use of fungicides for SR control was based on DMIs and their mixtures with QoIs. The DMIs alone showed high SR control, but their persistence period was shorter than when used in mixtures. In addition, they did not provide preventive action. When SR appeared in together with leaf rust (*Puccinia triticina*), DMIs were not efficient in controlling it because *P. triticina* has decreased sensitivity to this group of fungicides (Reis and Carmona, 2011). Likewise, field observations showed that DMIs can stop new SR infections, but cannot efficiently control infections older than 1 week or more, which will develop into necrotic stretch marks visually appearing as “a great necrotic area” (Carmona and Sautua, 2018). Therefore, mixtures of fungicide active ingredients proved to be a better option to SR control by increasing the period of protection, granting preventive and curative action while also being able to control both rusts with high efficiency (Reis and Carmona, 2011; Carmona et al., 2019).

## Optimal Fungicide Timing

Unlike other crop diseases, SR deserves special attention when deciding on fungicide application timing and frequency of reapplication. Being the most destructive disease of wheat, producers and government institutions should ensure that the use of fungicides minimizes losses, especially in susceptible varieties. It should also be remembered that in the current social and environmental context, the use of fungicides requires an in-depth analysis that ensures sustainability to avoid environmental damage and unnecessary applications while ensuring the sought profitability. For these reasons, the analysis, interpretation and the definition of the optimal fungicide timing for SR control is a relevant aspect.

The first need that must be met is related to the real and complete information of the sanitary reaction of commercial varieties. This information must be provided by the seed companies to the farmers (the latest information on the reactions of wheat genotypes to SR). This aspect is crucial because depending on the degree of susceptibility of the genotype there will or may not be a need to apply fungicides. The second aspect is to define whether or not it is convenient for scouting to be mandatory in wheat fields, since early detection of SR is a key factor to appropriately perform an on-time chemical control of the pathogen and gain maximum protection of yield. Timing of fungicide application can be critical for effective control of SR. The delay in the application of fungicides in relation to the exponential growth of SR epidemics have proved to be less profitable due to the losses caused by the disease (Jørgensen and Nielsen, 1994). Thus, crop scouting at least twice a week, beginning at tillering (GS 25, Zadoks et al., 1974) should be mandatory for successful early detection of the first SR pustules. Also, identifying “hot-spots” of infection and the use of disease trap nurseries may help in the early detection. These general guidelines need to be observed especially in the case of SR-susceptible cultivars (Chen and Kang, 2017b).

Another significant aspect is related to the information on the monitoring and regional traceability of fields being affected by SR. As it is known, the efficient capacity of *Pst* aerial dissemination over long distances is one of the most important causes for explaining the appearance of new diseased fields (Brown and Hovmøller, 2002). Therefore, knowledge of the occurrence of the disease in different regions and localities could be the basis for generating early warnings that help to strengthen and adjust scouting and be better prepared.

Additional information related to the availability and effectiveness of fungicides registered in each country is also required. In many cases, the sudden emergence of destructive SR epidemics caused by exotic *Pst* races has not given enough time to comply with the necessary regulations for the registration and assessment of fungicide efficacy. The 1961 SR epidemic in the USA Pacific Northwest can be mentioned as a historical example in which farmers had no available information on fungicides and had to apply, on an emergency basis, fungicides that had not been tested or registered in the nation (Line, 2002). Another current example is the SR epidemics that occurred in Argentina since 2017, after almost 90 years of little disease occurrence. In that case, in addition to the lack of knowledge on field disease diagnosis, producers and consultants had no technical guidance related to fungicide application timing, thus they had to resort to all types of fungicidal molecules to try to stop the epidemics (Carmona et al., 2019).

Information on fungitoxicity, dose and control efficiency of the different fungicidal molecules, together with all the aspects mentioned above, are essential to define with criteria the optimal time for SR chemical control.

According to Viljanen-Rollinson et al. (2002), a frequent question is to ask in what growth stages and with what disease intensity should the SR chemical control be carried out? The answer is not simple, it is rather complex and very difficult to harmonize. This is shown in the summary of the literature review regarding the recommendations for optimal fungicide timing for SR control (Table 2). The proposals are very diverse and based on different criteria not always coincident. Thus, for example, a frequent recommendation is to apply the fungicide “at first symptoms.” This proposal would not appear to be robust and should be analyzed in light of its practical significance. “First symptoms” means the first time a crop scout observes the symptoms in the field when scouting for SR. The first symptoms to be observed will depend on the interval or frequency with which each crop scout monitors. Therefore, if the scouting intervals between different crop scouts are different from each other, when comparing them, values of “first symptoms” of different intensity levels will be obtained depending on each visit frequency. While the main objective of scouting is to confirm the disease as early as possible, the disease onset data for a crop scout that visits a field plot twice a week will be different from another that performs a weekly inspection or every 10 days. It is very common that technical labels of commercial fungicides incorporate in their recommendation the phrase “apply to the first symptoms” without detailing levels of SR intensity or frequency of visits to wheat fields. The damage caused by SR depends on disease intensity and not on its presence—“first symptoms.”

**Table 2:** Fungicide timing recommended for SR control according to country and year.

**Country and region**	**Application timing**	**Year**	**References**
Argentina	10–20% SR incidence from stem elongation (GS32) (proposed as a preliminary guideline)	2017/2018	Carmona et al., 2019
Australia, Victoria	At seeding (applied on or adjacent to seed at sowing)	1983/1984	Brown et al., 1985
Australia, Victoria	If SR is present before ear emergence, spray before 1% severity (~35 leaves per 100 have stripe rust). When SR is first detected at ear emergence, only the most susceptible cultivars may need spraying.	2018	Hollaway, 2019
Australia, New South Wales	Spray when hot-spots are first seen, or when the incidence of stripe rust is 10–20 infected leaves per 100 green leaves.	2000s	Murray et al., 2005
Australia, Western Australia	If SR is present before ear emergence (GS59), then crops must be sprayed before the level of infection reaches 1% leaf area affected (this is when ~35 leaves per 100 have stripe rust).	2010s	McLean et al., 2010
Canada	Before all the heads had completely emerged (GS 55)	1984/1987	Conner and Kuzyk, 1988
China, south and east of Gansu Province	On seedlings before winter	–	Chen and Kang, 2017b
China	5–10% SR severity from flag leaves fully expanded to heading (GS49 to Z59)	2001/2002	Wan et al., 2004
China	Decisions about fungicide applications during grain filling based on knowledge of crop physiology. Application timing recommended: option (1) during grain filling; option (2) according to crop physiology.	2011/2013	He et al., 2019
Denmark	>1% plants with attack. GS 29–60 (S). >10% plants attacked after GS 61–71 (S)	Current recommendation	Eurowheat, 2020
Europe	At flag leaf emergence (GS 37–39)	2015/2016	Eurowheat, 2020
Finland	>1% plants with attack or foci (S) GS 29–59. >10% plants with attack (R)	Current recommendation	Eurowheat, 2020
France	From GS 31: at first symptoms. Before GS 31: if spots are present and they are active	Current recommendation	Eurowheat, 2020
Germany	First foci present	Current recommendation	Eurowheat, 2020
India, Punjab	Spray either at flag leaf emergence (GS 37–39) or when about 20% leaves showed SR symptoms	2010/2011, 2011/2012	Bal, 2014
India, New Delhi	At flag leaf emergence (GS 37–39)	2014/2015, 2015/2016	Singh et al., 2016
Italy	First symptom occurrence on the upper 2 leaves	Current recommendation	Eurowheat, 2020
Kenya	Two applications at tillering (GS 22) and flowering (GS 62)	2013 and 2014	Wanyera et al., 2016
Malaysia	Seed treatment and foliar spray applied at the 7 leaf stages	1985	Chan, 1985
Netherlands	At first symptoms	Current recommendation	Eurowheat, 2020
New Zealand	Stem elongation (GS32)/awn emergence (GS59)	1980s	Gaunt and Cole, 1991
Poland	At GS 30–31: 25–30% tillers with lesions	Current recommendation	Eurowheat, 2020
South Africa	Seed treatment combined with foliar fungicides	1990s	Boshoff et al., 2003
South Africa	Seven leaves unfolded (GS 16–19)/awns visible to emergence of spike completed = flag leaf (GS 49–59)	1990s	Boshoff et al., 2003
USA	Mixing fungicide with herbicide at the time of herbicide application if needed and apply fungicide at flag-leaf stage if necessary.	Current recommendation	Chen and Kang, 2017b
USA, Nebraska	At first detection of SR in the field if the following conditions are met: (1) SR-favorable weather (cool, wet conditions) is forecast, (2) the flag leaf has emerged, (3) SR has been confirmed in southern states, and (4) SR has been detected in the field. If disease pressure is heavy in southern states and SR appears earlier than flag leaf emergence, a pre-flag leaf fungicide application may be warranted followed by a flag leaf application.	2012	Jackson-Ziems et al., 2016
USA, Nebraska	At flag leaf emergence (GS 37–39)	2015	Wegulo, 2015
USA, Montana	At flag leaf emergence (GS 37–39)	2016	Turner et al., 2016
United Kingdom	Seed treatment	1989, 1990	Cheer et al., 1990
United Kingdom	Period between flag-leaf emergence (GS 37–39) and early ear emergence (GS 39–55)	1988/1990	Hims and Cook, 1991
United Kingdom	1–2% severity or foci present	Current recommendation	Eurowheat, 2020
Uruguay	At first symptoms	Current recommendation	Germán et al., 2018

Another frequent recommendation is based on the wheat growth stage as an indicator of fungicide timing (Table 2). Under this recommendation, the idea of prioritizing its effects on the host prevails over the pathogen. There is a general acceptance among producers and consultants that the main objective of a fungicide is to “protect and cure the host” without taking into account its action on pathogens. In this way, many fungicides are applied depending on the crop phenological stage with the mission that the upper leaves, mainly involved in the generation of grain yield, receive the chemical. There are numerous works that indicate the need to protect the upper leaves (Poole and Arnaudin, 2014). According to Viljanen-Rollinson et al. (2002), the recommendation to apply fungicides for the SR control should be made during the emergence of the flag leaf (GS 39). In coincidence, De Wolf et al. (2012) mention that a fungicide generates less SR control and consequently gives less yield response when applied before the emergence of the flag leaf. In this phyto-centric vision, the lower leaves that do not contribute to yield are not taken into account, even if they are diseased. In this framework of analysis, it is highly probable that, for example, when the fungicide is applied SR incidence and severity in the lower leaves are high and thus will be the main multipliers of the disease in the field plot. Undoubtedly, both the SR control and economic responses will be reduced if chemical applications are delayed. This discussion becomes even more relevant when considering the epidemiology of SR, a polycyclic disease that depends fundamentally on the rate of development. The number of sporulant uredinia in the lower leaves are a measure of importance of the transfer of inoculum toward the upper leaves. Thus, for example, Young et al. (2003) developed in England a model of prediction of the growth of the disease in a field plot based exclusively on lower leaf infections. These observations are in line with the assessments of Braithwaite et al. (1998), who indicate that the time of SR onset is the most critical factor in defining the time of fungicide application, rather than the phenological stage *per se*. When the disease was observed after heading, the application that provided the greatest control of SR was performed at flowering (GS 62), compared with the application guided only by the growth stage (fixed) in stem elongation (GS 32).

Although understanding the physiology of the host and the phenological stages is relevant, it should not be the only information for guiding fungicide applications for SR control. For all this, it is important to consider the role of diseased lower leaves (especially in very susceptible varieties). Lower leaves may contribute less to yield grain formation but contribute to the multiplication and spread of the pathogen. It is necessary to stop the *Pst* high rate of multiplication at the beginning of an epidemic that may not coincide with the time of the appearance of the upper leaves. This criterion coincides with Burkow et al. (2014), who mentions that fungicide applications at the early stages of SR infection are much more effective and can stop subsequent reinfections. The smaller the population of the pathogen to be controlled, the greater the control and persistence of the fungicide. When fungicides are applied opportunely to infections in lower leaves and even without having appeared the upper leaves, there is a decrease in the inoculum present in the field plot. This is an effective way of exercising an “indirect protection” on leaves that have not yet emerged because the inoculum is reduced in the field plot. In general, the initial or primary inoculum of rusts is brought in fields by wind, but the multiple reinfections within a field are mainly due to previously infected leaves, predominantly, the lower ones within the canopy (Farber, 2017).

At the opposite end of this analysis are the recommendations that indicate applying fungicides under a “preventive” scheme (Boshoff et al., 2003), that is, when wheat plants still do not show symptoms or visual signs. In these cases, the results are erratic and even fungicide applications for SR control could result in negative economic returns if there is no infection (Burkow et al., 2014). In this line of research, Viljanen-Rollinson et al. (2010) reported that when susceptible cultivars were sowed the impact of fungicides applied preventively during stem elongation (GS 31 and 37) was more effective in controlling SR vs. if they were applied at the first symptoms. The opposite happened when it came to varieties with some resistance (partial resistance). The authors concluded that preventive applications carried out in a fixed growth stage in the absence of the disease, were the most suitable for very susceptible cultivars, while the application performed at first symptoms could be a more appropriate method in those cultivars that slow down the disease because of having a certain genetic resistance, such as being moderately resistant.

Other recommendations when making fungicide application decisions for SR control are related to the economic damage threshold (EDT). Some authors established levels of foliar incidence and/or severity from which the economic return of the fungicide application is justified (Brown and Holmes, 1983; Murray, 2004; Wan et al., 2004; Murray et al., 2005; McLean et al., 2010; Bal, 2014; Jørgensen et al., 2014; Carmona and Sautua, 2018; Hollaway, 2019). These SR intensity thresholds are in the approximate range of 10–35% of leaf incidence (10–35 leaves per 100 have stripe rust) (Table 2). In order to guide fungicide applications using this type of technical recommendation, scouting is essential and must be continued even after applying the fungicide. This ensures that if there is reinfection, a new fungicide application can be carried out on time. The main drawback of this type of threshold is that the disease, unlike other rusts, can frequently appear in the field plots as hot spots or “foci.” Therefore, scouting should be directed to those spots or be exhaustive throughout the whole field, examining as many plants as possible.

Other authors have developed prediction models to help define the most appropriate fungicide timing for SR control. For example, in Australia, Brown and Holmes (1983) determined the different infection rates in different wheat cultivars and with a statistical prediction model they adjusted the best time for fungicide use. Eddy (2009) modeled the probability of wheat yield response in Kansas, USA, based on disease resistance of a variety, historical disease risk, and in-season disease risk with an accuracy between 71 and 84%. According to Chen and Kang (2017b), the optimal fungicides timing is not fixed and varies fundamentally according to the moment of occurrence of the disease in the crop, the cultivar involved, the environment, the region considered and the economic variables at stake.

Defining the optimal fungicide application timing for SR control is not a simple task and it should not be performed based on a single criterion. On the contrary, such complexity must be analyzed carefully. Decision-making necessarily forces us to consider various aspects in an integrated manner, including the host, the epidemiology of the disease, the fungicides and economic variables. It is necessary to analyze the attributes of the pathogen (polycyclic, races, inoculum pressure); the host (critical period for grain yield determination, cultivar degree of susceptibility, potential yield); the fungicide (dose, type of molecule), the environment (dew, rain, temperature, foliar wetness, relative humidity); and the presence of other diseases besides SR along with the analysis of disease economic return.

In relation to the fungicidal active ingredients, it is important that they ensure both preventive and curative action. Therefore, mixtures of SDHI or QoI plus DMIs are ideal for efficiently meeting these objectives. Another important aspect is to respect not only the optimal moment of control but also the recommended doses. Thus, for example, Jørgensen and Nielsen (1994) found that low DMI dosages applied on susceptible varieties had a shorter protection period than full dose when SR outbreaks (high disease pressure) have occurred in Denmark, requiring additional treatments. However, Sharma et al. (2016) showed that lower concentrations of different fungicides could offer good control and be profitable when the disease pressure is not very high.

## Number of Fungicide Applications

In general, the number of fungicide applications needed to control SR depends on the inoculum pressure, the level of resistance of the sown cultivars, the timing of disease appearance in the field, and the occurrence of an environment predisposing SR epidemic development. For example, in Argentina, two applications of a QoI + DMI mixture (Carmona et al., 2019), on SR resistant varieties showed a negative economic impact, thereby suggesting that the applications were unnecessary. On the other hand, on SR susceptible varieties, one or two applications significantly reduced the intensity of the disease and protected yields. Moreover, two applications offered a significantly higher yield return in comparison to a single application. The crop season during which those studies took place was characterized by a high SR pressure and a predisposing environment to the reinfections of field plots previously sprayed with fungicide. In most of the wheat varieties evaluated, double application was justified.

## Wheat Yield Response to Fungicide Applications

Several reports illustrate the variability among wheat yield response to fungicides when applied to control SR. Those values are dependent on multiple factors, such as type of fungicide, application timing, number of applications, predominant *Pst* races, wheat variety's degree of susceptibility to SR, environmental variables, and application technology, to mention a few. However, it is clear that in all cases in which epidemics of SR occurred, the impact of fungicides was significant, allowing to reduce or avoid losses and generate higher yields both in number and weight of grains and in quality of wheat harvested. Thus, only by way of example, in Denmark, Jørgensen and Nielsen (1994) reported and average yield increase of 17, 30, 53, and 68% with one, two, three or four applications of a DMI + morpholine mixture (ergosterol inhibitors) using different doses and timing. In South Africa, Boshoff et al. (2003) reported about increases in yields as high as 49% under predisposing conditions for SR. In Canada, Xi et al. (2015) showed in field trials that the application of fungicides increased yield between 15 and 23% and thousand kernel weight (TKW) by 8–10%. In the USA, the use of fungicides to control SR allowed to reduce important yield losses. For example, Chen (2007) reported that the use of fungicides in Washington State alone allowed farmers to save 15–30 million dollars from 2002 to 2005. In another state, Texas, the chemical application resulted in yield increases up to 41% and in the TKW up to 33% (Reid and Swart, 2004). Chen et al. (2016) evaluated 24 wheat varieties in a field near Pullman, WA, in 2015 and reported that fungicide application resulted in yield increases from −4.26 to 38.18%. Sharma et al. (2016) estimated after 2 years in 3 locations in Tajikistan and Uzbekistan an average grain yield increase of 44 and 48% with a single or two fungicide applications, respectively. In India, Singh et al. (2016) evaluated during 2 growing seasons different fungicides applied in different growth stages. These researchers reported average yield increases of 22.8, 81.9, 61, and 39% when the application was made at early stem extension, flag leaf emergence, booting and heading, respectively. Also, Ahanger et al. (2014) reported an average yield increase of 44 and 29.8% in SR-susceptible and resistant wheat varieties, respectively. Jørgensen et al. (2018) conducted a study that involved 40 field trials during two seasons (2015 and 2016) in 10 different countries across Europe and tested four DMI fungicides, alone or in mixtures of two active ingredients at different field doses. These researchers reported grain yield increases between 13 and 44% depending on the dose and the year of evaluation. In Argentina, Carmona et al. (2019) recently reported yield responses to a QoI+DMI mixture varied from 0 to 158% and 5 to 242% for one and two sprays, respectively, depending on the resistance level of the variety evaluated. The evaluations were carried out in fields severely affected by epidemics of the newly introduced exotic race *PstS13*.

## Fungicide Resistance Management

Fungicide resistance is a term that refers to an acquired and inheritable reduction in the sensitivity of a fungus to a specific antifungal active substance (Beckerman, 2013). In recent years, several cases of fungicide resistance in plant pathogenic fungi have been increasingly reported worldwide (Hollomon, 2015). Therefore, pathogen resistance to different fungicide active ingredients is one of the most important aspects in current agriculture (Lucas et al., 2015).

In some cases, fungicide resistance can appear very strongly, quickly and in a single step, as in the case of the G143A mutation (the substitution of alanine for glycine at codon 143 in the mitochondrial cytochrome b gene) that affects the QoIs (Gisi et al., 2002). In other cases, the emergence of resistance may be gradual and the loss of sensitivity of the pathogen is progressive, as is the case of resistance to DMIs (Brent and Hollomon, 2007). In general, the resistance risk level of rust fungi to QoIs is low and even lower to DMIs (Oliver, 2014). Thus, by way of example, the resistance to QoI generated by the G143A mutation has never been observed in any species of rust (Brent and Hollomon, 2007). Currently, SR control efficacy by fungicides is high despite the increased use prompted by the occurrence of severe epidemics worldwide (Singh et al., 2016; Chen et al., 2018; Jørgensen et al., 2018).

Although there have been reports of sensitivity reduction throughout the history of its use (Bayles et al., 2000; Napier et al., 2000), in recent years no cases of control failure in SR-infected field plots treated with fungicides nor detection of fungicide resistance produced artificially in the laboratory have been reported, except for a single report of loss of sensitivity to triadimefon (Tian et al., 2019). Kang et al. (2019) monitored the sensitivity of several *Pst* isolates from the United States and demonstrated that although propiconazole and pyraclostrobin fungicides are still efficient, there are differences in sensitivity in isolates. Recently, Peng et al. (2020) developed a rapid method of quantifying fungicide effectiveness against *Pst* with detached leaves. They propose this method could be used for determining effective concentration (EC_50_) values for fungicides and test the sensitivity of different *Pst* isolates.

Due to intense use of fungicides in the past, the risk of resistance development must be taken into account as a priority to develop sustainable chemical control strategies. Therefore, it is extremely important that producers and consultants correctly use different fungicide active ingredients to enhance their efficacy and prolong their useful lifespan as many years as possible. The following approaches should be employed with a holistic or integrated approach: (1) use the genetic resistance of the wheat varieties available in each producing region as the first component in SR management; (2) plan and implement an integrated disease management (IDM) program that includes: use of pathogen-free seed, crop scouting, disease monitoring, crop rotation, and application of other cultural practices, such as nutrition diagnosis and water management; (3) apply a fungicide only when necessary at the optimal application timing defined according to the available scientific methodology; (4) use mixtures of fungicide active ingredients with different biochemical mechanism of action. Each active ingredient must have high efficiency in the control of SR; (5) alternate fungicide active ingredients (between and within the same biochemical mechanism of action); (6) complement fungicides with resistance inducers and/or biological control agents; (7) follow the labeled doses of commercial fungicides indicated by the manufacturers and obey the restrictions indicated therein; (8) develop a program to monitor the sensitivity of *Pst* populations.

## Integrated Control of SR

**Figure 4:**
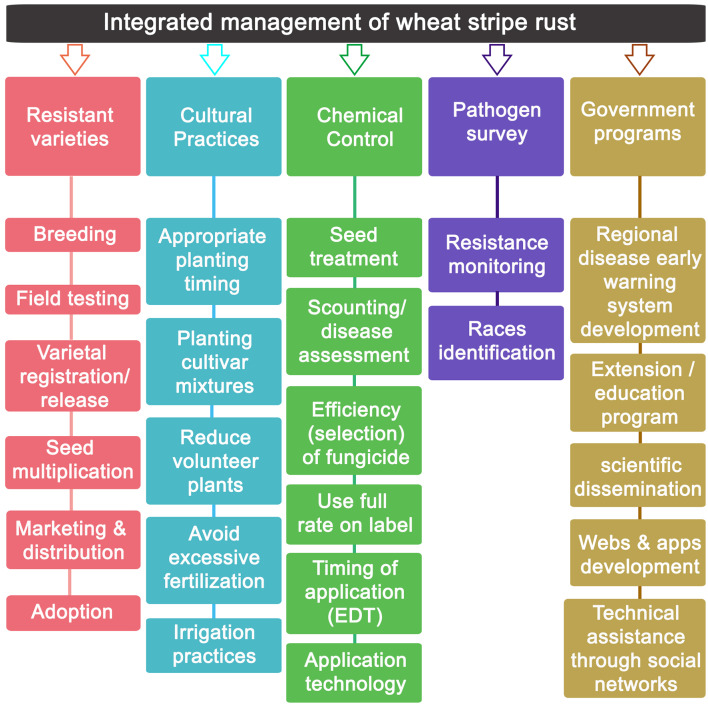
Integrated management and prevention of wheat stripe rust scheme. An integrated stripe rust management approach is based on the combination of crop cultivars with adequate levels of resistance, use of early-warning systems involving regular pathogen monitoring and disease scouting, cultural practices, and timely application of fungicides. The base figure is modified from FAO (2014).

An integrated SR management program combines the use of crop cultivars with adequate levels of resistance, use of early-warning systems involving regular pathogen monitoring and disease scouting, cultural practices and timely application of effective fungicides (Figure 4) (Line, 1993; Chen et al., 2013; Chen and Kang, 2017b). Control of SR should start with the development of wheat varieties with appropriate and durable levels of resistance. However, because there are no cultivars that are resistant to all *Pst* races (Zeng et al., 2014), the use of fungicides has become one of the most important practices for SR control worldwide.

Economic, environmental, and legal factors influence fungicide selection and application timing. Until now, fungicides have shown to be significant in reducing SR intensity and yield losses and therefore have become practically the only control option in non-resistant varieties of wheat. Historically, fungicides have allowed us to avoid large economic losses that would have occurred due to the SR epidemics (Line, 2002; Chen and Kang, 2017b). Therefore, it is affirmed that at present it is almost impossible to plant SR-susceptible varieties of wheats without the need to apply fungicides in regions where the disease is endemic. For that reason, and despite their possible negative impacts on the environment, fungicides will continue to be very useful in SR management due to their high effectiveness in defending against SR and relatively low cost.

## Conclusion and Perspectives

The SR remains a major phytosanitary challenge to wheat cultivation and a threat to wheat production worldwide; its global economic impact remains elusive. Our analysis suggests that future SR epidemics are likely to occur worldwide due to: (i) the presence and emergence of aggressive well-adapted *Pts* strains that can cause severe epidemics in different wheat growing regions, (ii) the limited availability of wheat cultivars with medium to high SR resistance levels and high yield response, and (iii) the likelihood of more frequent extreme weather events that are conducive to long-distance dispersal of rust spores. In view of that, we believe that fungicides will continue to play a key role in SR management, even when wheat cultivars with high level of resistant to the greatest possible number of *Pst* races become available.

There is limited information on the performance and economic impact of fungicides in controlling SR worldwide. Yet, the effectiveness of QoIs and DMIs is well-proven. The efficacy and protection period of the recently introduced SDHIs still needs to be evaluated. Availability of other products, such as defense activators or biological control agents, alone or in combination with fungicides, will also be crucial to SR management programs.

Within fungicide use, a problem that requires further research is the definition of an optimal fungicide application timing. This will avoid unnecessary applications, thereby minimize negative environmental and social impact. We propose the use of an economic damage threshold (EDT) as the basis for a decision support tool for timing fungicide applications. Ideally, the development and validation stages of the EDT should be carried out locally for at least 3 to 5 growing seasons. Field experiments should include both the most popular wheat varieties and the most used fungicides in each region. Since a good relationship between rust severity and incidence is generally observed then the EDT can use incidence as the preferred disease measurement. Incidence is easier to determine than severity in the field and more practical for researchers and farmers. The EDTs could be integrated with weather forecasts into complex models and thereafter into operational warning systems to predict the probability of crop yield response to a fungicide/s application. Nevertheless, it is important to understand that in field conditions several wheat diseases most often coexist and interact. This problem has been faced by farm advisers when defining the optimal timing for the first fungicide application in situations where more than one foliar disease co-occur on the same crop. Therefore, a multi-disease approach is essential.

It is unknown whether the widespread fungicide use will quickly generate selection pressure on less sensitive isolates in the *Pst* population and if that could render fungicides less effective. This is a fundamental point that must not be overlooked, and that future research should address. Fungicide resistance monitoring programs should be part of the SR integrated management. Therefore, it is always necessary to reevaluate fungicides annually due to: (i) fungicide sensitivity shifts in different SR strains; (ii) the development of fungicide resistance in pathogen populations; (iii) the appearance of new SR races; and (iv) the necessity to know the reaction of different cultivars.

Fungicide application technology also plays a role in control efficacy. There are practically no modern studies in this regard and is another aspect that research should address.

In some countries, there is still need to establish official survey teams that help keep regional wheat producers informed about SR occurrence and spread. Also, training on scouting and diagnosis should be imparted in some countries to growers and field managers, since reemergence of SR, after a long time of being unseen, is practically a new problem.

In summary, success in controlling SR should start with pathogen surveys and the development of an IDM program that includes use of wheat varieties with high level of SR resistance and correct use of fungicides. The implementation of an IDM program should rely on early disease warning systems developed from regular pathogen scouting and EDTs, thus SR epidemics are predicted with high level of confidence.

## Author Contributions

MC, FS, and OP-H wrote the manuscript. ER revised the manuscript. All authored reviewed and contributed to the manuscript.

## Conflict of Interest

The authors declare that the research was conducted in the absence of any commercial or financial relationships that could be construed as a potential conflict of interest.

## References

[B1] Agrios G. (2005). Plant Pathology. 5th Edn. Amsterdam: Elsevier Academic Press.

[B2] Ahanger R. A. Gupta V. Bhat H. A. Dar N. A. (2014). Management of yellow rust (*Puccinia striiformis* West) of wheat and its impact on yield under Jammu sub-tropics of India. Bioscan 9, 215–218.

[B3] AHDB (2019). “Fungicide performance update for wheat, barley, and oilseed rape 2019,” in AHDB Agronomists' Conference, December 3–4 2019 (Leicester: Agriculture and Horticulture Development Board). Available online at: https://projectblue.blob.core.windows.net/media/Default/Imported%20Publication%20Docs/AHDB%20Cereals%20&%20Oilseeds/Disease/Fungicide%20performance/Fungicide%20performance%202019%20(6.12.19).pdf (accessed February 1, 2020).

[B4] Ali S. Gladieux P. Leconte M. Gautier A. Justesen A. F. Hovmøller M. S.. (2014a). Origin, migration routes and worldwide population genetic structure of the wheat yellow rust pathogen *Puccinia striiformis* f.sp. *tritici*. PLoS Pathog. 10:e1003903. 10.1371/journal.ppat.1003903 24465211 PMC3900651

[B5] Ali S. Gladieux P. Rahman H. Saqib M. S. Fiaz M. Ahmad H.. (2014b). Inferring the contribution of sexual reproduction, migration and off-season survival to the temporal maintenance of microbial populations: a case study on the wheat fungal pathogen *Puccinia striiformis* f.sp. *tritici*. Mol. Ecol. 23, 603–617. 10.1111/mec.12629 24354737

[B6] Ali S. Leconte M. Rahman H. (2014c). A high virulence and pathotype diversity of *Puccinia striiformis* f.sp. *tritici* at its centre of diversity, the Himalayan region of Pakistan. Eur. J. Plant Pathol. 140, 275–290. 10.1007/s10658-014-0461-2

[B7] Ali S. Rodriguez-Algaba J. Thach T. Sørensen C. K. Hansen J. G. Lassen P.. (2017). Yellow rust epidemics worldwide were caused by pathogen races from divergent genetic lineages. Front. Plant Sci. 8:1057. 10.3389/fpls.2017.01057 28676811 PMC5477562

[B8] Amaro A. C. E. Baron D. Ono E. O. Rodrigues J. D. (2019). Physiological effects of strobilurin and carboxamides on plants: an overview. Acta Physiol. Plant. 42:4 10.1007/s11738-019-2991-x

[B9] APVMA (2015). Fluxapyroxad in the Product Systiva Seed Treatment Fungicide. Australian Pesticides and Veterinary Medicines Authority. Available online at: https://apvma.gov.au/node/13241 (accessed February 1, 2020).

[B10] ARC Small Grain Institute (2014). More on the Chemical Control of Wheat Rust. Available online at: https://www.grainsa.co.za/more-on-the-chemical-control-of-wheat-rust (accessed February 1, 2020).

[B11] Ash G. J. Brown J. F. (1990). Yield losses in wheat caused by stripe rust (*Puccinia striiformis* West.) in northern New South Wales. Aust. J. Exp. Agric. 30, 103–108.

[B12] Bal R. S. (2014). Effect of some fungicides and time of fungicidal spray on stripe rust of wheat. J. Plant Pest Sci. 1, 39–43.

[B13] Balba H. (2007). Review of strobilurin fungicide chemicals. J. Environ. Sci. Health B 42, 441–451. 10.1080/03601230701316465 17474024

[B14] Bartlett D. W. Clough J. M. Godwin J. R. Hall A. A. Hamer M. Parr-Dobrzanski B. (2002). The strobilurin fungicides. Pest Manag. Sci. 58, 649–662. 10.1002/ps.520 12146165

[B15] Batts C. C. V. Elliott C. S. (1952). Indications of effects of yellow rust on yield of wheat. Plant Path. 1, 130–131.

[B16] Bayles R. A. Stigwood P. L. Clarkson J. D. S. (2000). Shifts in sensitivity of *Puccinia striiformis* DMI fungicides in the UK. Acta Phytopathol. Entomol. Hungarica 35, 381–382.

[B17] Beckerman J. L. (2013). “Detection of fungicide resistance,” In: *Fungicides–Showcases of Integrated Plant Disease Management From Around the World*, ed N. Nita (London: InTech). 10.5772/55981 Available online at: https://www.intechopen.com/books/fungicides-showcases-of-integrated-plant-disease-management-from-around-the-world/detection-of-fungicide-resistance (accessed February 1, 2020).

[B18] Beddow J. M. Pardey P. G. Chai Y. Hurley T. M. Kriticos D. J. Braun H.-J, Park, R. F.. (2015). Research investment implications of shifts in the global geography of wheat stripe rust. Nat. Plants 1:15132. 10.1038/nplants.2015.132 27251389

[B19] Beresford R. M. (2011). Succinate Dehydrogenase Inhibitor (SDHI) Fungicide Resistance Prevention Strategy. Available online at: https://resistance.nzpps.org/index.php?p=fungicides/sdhi (accessed February 1, 2020).

[B20] Bever W. M. (1937). Influence of stripe rust on growth, water economy and yield of wheat and barley. J. Agric. Res. 54, 375–385.

[B21] Boshoff W. H. P. Pretorius Z. A van Niekerk B. D (2003). Fungicide efficacy and the impact of stripe rust on spring and winter wheat in South Africa. S. Afr. J. Plant Soil 20, 11–17. 10.1080/02571862.2003.10634898

[B22] Braithwaite M. Cromey M. Saville D. Cookson T. (1998). “Effects of fungicide rates and timing on control of stripe rust in wheat,” in Proceedings of the 51st New Zealand Plant Protection Conference (Hamilton), 66–70. 10.30843/nzpp.1998.51.11651

[B23] Brent K. J. Hollomon D. W. (2007). Fungicide Resistance: The Assessment of Risk. FRAC Monograph 2. 2nd Edn. Brussels: FRAC Available online at: http://www.frac.info/docs/default-source/publications/monographs/monograph-2.pdf (accessed February 1, 2020).

[B24] Brown J. K. Hovmøller M. S. (2002). Aerial dispersal of pathogens on the global and continental scales and its impact on plant disease. Science. 297, 537–541. 10.1126/science.1072678 12142520

[B25] Brown J. S. Ballinger D. J. Kollmorgen J. F. (1985). Effects of fungicides applied at seeding on stripe rust and common bunt of wheat. Crop Prot. 4, 481–484. 10.1016/0261-2194(85)90053-5

[B26] Brown J. S. Holmes R. J. (1983). Guidelines for use of foliar sprays to control stripe rust of wheat in Australia. Plant Dis. 67, 485–487.

[B27] Bryson R. J. Paveley N. D. Clark W. S. Sylvester-Bradley R. Scott R. K. (1997). Use of in-field measurements of green leaf area and incident radiation to estimate the effects of yellow rust epidemics on the yield of winter wheat. Dev. Crop Sci. 25, 77–86. 10.1016/S0378-519X(97)80010-4

[B28] Buendía-Ayala B. L. Martínez-Cruz E. Villaseñor H. E. Hortelano Santa Rosa R. Espitia-Rangel E. Buendía-González M. O. (2019). The incidence of yellow rust and the industrial quality of the grain and the dough in bread wheat. Rev. Mexicana Cienc. Agric. 10, 143–154. 10.29312/remexca.v10i1.1333

[B29] Burkow J. Singh A. K. Valle V. Velázquez J. C. Padilla D. P. Renova J. (2014). A Model for Stripe Rust Growth With Two Fungicidal Effects. Available online at: https://mtbi.asu.edu/2014-4 (accessed February 1, 2020).

[B30] Cantu D. Segovia V. MacLean D. Bayles R. Chen X. Kamoun S.. (2013). Genome analyses of the wheat yellow (stripe) rust pathogen *Puccinia striiformis* f. sp. *tritici* reveal polymorphic and haustorial expressed secreted proteins as candidate effectors. BMC Genomics 14:270. 10.1186/1471-2164-14-270 23607900 PMC3640902

[B31] Carmona M. Sautua F. (2018). Epidemias de roya amarilla del trigo. nuevas razas en el mundo, monitoreo y decisión de uso de fungicidas. Agron. Ambiente Rev. Facult. Agron. UBA 38, 37–58.

[B32] Carmona M. A. Sautua F. J. Pérez-Hernández O. (2019). Rapid emergency response to yellow rust epidemics caused by newly introduced lineages of *Puccinia striiformis* f. sp. *tritici* in Argentina. Trop. Plant Pathol. 44:385 10.1007/s40858-019-00295-y

[B33] Chan K. C. (1985). “Timing of fungicide applications for disease control in wheat,” in Proceedings of the Regional Conference on Plant Quarantine Support for Agricultural Development. Kuala Lumpur, Malaysia, 10–12 December 1985 (Serdang: ASEAN Plant Quarantine Centre and Training Institute), 125–129.

[B34] Chaves M. S. Martinelli J. A. Wesp-Guterres C. Graichen F. A. S. Brammer S. P. Scagliusi S. M. (2013). The importance for food security of maintaining rust resistance in wheat. Food Sec. 5:157 10.1007/s12571-013-0248-x

[B35] Cheer A. B. Heatherington P. J. Clark D. C. (1990). “Control of yellow rust with a triadimenol seed treatment on a range of winter wheat cultivars,” in Brighton Crop Protection Conference, Pests and Diseases−1990 (Brighton), 807–812.

[B36] Chen W. Wellings C. Chen X. Kang Z. Liu T. (2014). Pathogen profile: wheat stripe (yellow) rust caused by *Puccinia striiformis* f. sp. *tritici*. Mol. Plant Pathol. 15, 433–446. 10.1111/mpp.12116 24373199 PMC6638732

[B37] Chen W. Q. Kang Z. S. Ma Z. H. Xu S. C. Jin S. L. Jiang Y. Y. (2013). Integrated management of wheat stripe rust caused by *Puccinia striiformis* f. sp. *tritici* in China. Sci. Agric. Sin. 46, 4254–4262. 10.3864/j.issn.0578-1752.2013.20.008

[B38] Chen W. Q. Wu L. R. Liu T. G. Xu S. C. Jin S. L. Peng Y. L.. (2009). Race dynamics, diversity, and virulence evolution in *Puccinia striiformis* f. sp. *tritici*, the causal agent of wheat stripe rust in China from 2003 to 2007. Plant Dis. 93, 1093–1101. 10.1094/PDIS-93-11-1093 30754577

[B39] Chen W. Q. Xu S. C. Wu L. R. (2007). Epidemiology and sustainable management of wheat stripe rust caused by *Puccinia striiformis* West. in China: a historical retrospect and prospect. Sci. Agric. Sin. 40, 177–183.

[B40] Chen X. M. (2005). Epidemiology and control of stripe rust [*Puccinia striiformis* f. sp. *tritici*] on wheat. Can. J. Plant Pathol. 27, 314–337. 10.1080/07060660509507230

[B41] Chen X. M. (2007). Challenges and solutions for stripe rust control in the United States. Aust. J. Agric. Res. 58, 648–655. 10.1071/AR07045

[B42] Chen X. M. (2014). Integration of cultivar resistance and fungicide application for control of wheat stripe rust. Can. J. Plant Pathol. 36, 311–326. 10.1080/07060661.2014.924560

[B43] Chen X. M. (2020). Pathogens which threaten food security: *Puccinia striiformis*, the wheat stripe rust pathogen. Food Sec. 12, 239–251. 10.1007/s12571-020-01016-z

[B44] Chen X. M. Evans C. K. Liu Y. M. (2016). Responses of winter wheat cultivars to fungicide application for control of stripe rust in 2015. Plant Dis. Manage. Rep. 10:CF023.

[B45] Chen X. M. Evans C. K. Sprott J. Liu Y. M. (2018). Evaluation of foliar fungicide treatments for control of stripe rust on winter wheat in 2017. Plant Dis. Manage. Rep. 12:CF073.

[B46] Chen X. M. Kang Z. (2017a). “Introduction: history of research, symptoms, taxonomy of the pathogen, host range, distribution, and impact of stripe rust,” in Stripe Rust, eds X. Chen and Z. Kang (Dordrecht: Springer), 1–33. 10.1007/978-94-024-1111-9_1

[B47] Chen X. M. Kang Z. (2017b). “Integrated control of stripe rust,” in Stripe Rust, eds X. Chen and Z. Kang (Dordrecht: Springer), 559–599. 10.1007/978-94-024-1111-9_6

[B48] Chen X. M. Penman L. Wan A. M. Cheng P. (2010). Virulence races of *Puccinia striiformis* f. sp. *tritici* in 2006 and 2007 and development of wheat stripe rust and distributions, dynamics, and evolutionary relationships of races from 2000 to 2007 in the United States. Can. J. Plant Pathol. 32, 315–333. 10.1080/07060661.2010.499271

[B49] Chen Y. L. Xie S. X. Sun Y. H. Qin H. K. (1982). Preliminary research on triadimefon as a seed dressing for controlling epidemics of yellow rust of wheat. Chin. J. Plant Protec. 9, 265–270.

[B50] Chen Y. L. Xie S. X. Sun Y. H. Qin H. K. (1984). Preliminary research on the control of stripe rust (*Puccinia striiformis*) of wheat by triadimefon. Chin. J. Plant Protec. 11, 241–246.

[B51] Cheng Y. Yao J. Zhang Y. Li S. Kang Z. (2016). Characterization of a Ran gene from *Puccinia striiformis* f. sp. *tritici* involved in fungal growth and anti-cell death. Sci. Rep. 6:35248. 10.1038/srep35248 27734916 PMC5062253

[B52] Conner R. L. Kuzyk A. D. (1988). Effectiveness of fungicides in controlling stripe rust, leaf rust, and black point in soft white spring wheat. Can. J. Plant Pathol. 10, 321–326. 10.1080/07060668809501706

[B53] Cools H. J. Hawkins N. J. Fraaije B. A. (2013). Constraints on the evolution of azole resistance in plant pathogenic fungi. Plant Pathol. 62, 36–42. 10.1111/ppa.12128

[B54] Cuomo C. A. Bakkeren G. Khalil H. B. Panwar V. Joly D. Linning R.. (2017). Comparative analysis highlights variable genome content of wheat rusts and divergence of the mating loci. G3-Genes Genom. Genet. 7, 361–376. 10.1534/g3.116.032797 27913634 PMC5295586

[B55] de Vallavieille-Pope C. Bahri B. Leconte M. Zurfluh O. Belaid Y. (2018). Thermal generalist behaviour of invasive *Puccinia striiformis* f. sp. *tritici* strains under current and future climate conditions. Plant Pathol. 67, 1307–1320. 10.1111/ppa.12840

[B56] de Vallavieille-Pope C. Huber L. Leconte M. Goyeau H. (1995). Comparative effects of temperature and interrupted wet periods on germination, penetration, and infection of *Puccinia recondita* f. sp. *tritici* and *P. striiformis* on wheat seedlings. Phytopathol. 85, 409–415. 10.1094/Phyto-85-409

[B57] De Wolf E. Bockus W. Shoup D. Eddy R. Duncan S. Shroyer J. (2012). Evaluating the Need for Wheat Foliar Fungicides. Agricultural Experiment Station and Cooperative Extension Service, Kansas State University. Available online at: https://www.bookstore.ksre.ksu.edu/pubs/MF3057.pdf (accessed February 1, 2020).

[B58] Doodson J. K. Manners J. G. Myers A. (1964b). Some effects of yellow rust (*Puccinia striiformis*) on the growth and yield of a spring wheat. Ann. Bot. 28, 459–472.

[B59] Doodson J. K. Manners J. G. Myers A (1964a). “Some effects of yellow rust on the yield and physiology of wheat,” in Paper Presented at the Third International Yellow Rust Conference (Cambridge).

[B60] Eddy R. (2009). Logistic regression models to predict stripe rust infections on wheat and yield response to foliar fungicide application on wheat in Kansas (M. Sc. Thesis), B. S. Kansas State University, Manhattan, KS, United States. Available online at: https://core.ac.uk/download/pdf/5166244.pdf (accessed February 1, 2020).

[B61] El Jarroudi M. Kouadio L. Bock C. H. El Jarroudi M. Junk J. Pasquali M.. (2017). A threshold-based weather model for predicting stripe rust infection in winter wheat. Plant Dis. 101, 693–703. 10.1094/PDIS-12-16-1766-RE 30678577

[B62] Elbasyoni I. S. El-Orabey W. M. Morsy S. Baenziger P. S. Al Ajlouni Z. Dowikat I. (2019). Evaluation of a global spring wheat panel for stripe rust: Resistance loci validation and novel resources identification. PLoS ONE 14:e0222755. 10.1371/journal.pone.0222755 31721783 PMC6853611

[B63] Ellis J. G. Lagudah E. S. Spielmeyer W. Dodds P. N. (2014). The past, present and future of breeding rust resistant wheat. Front. Plant Sci. 5:641. 10.3389/fpls.2014.00641 25505474 PMC4241819

[B64] Eurowheat (2020). Control Thresholds for Diseases. Available online at: https://agro.au.dk/forskning/internationale-platforme/eurowheat/wheat-ipm-tools-and-information/control-thresholds-for-diseases/ (accessed February 1, 2020).

[B65] FAO (2014). Wheat Rust Diseases Global Programme 2014–2017. Food and Agriculture Organization of the United Nations. Available online at: http://www.fao.org/3/a-i3730e.pdf (accessed February 1, 2020).

[B66] Farber D. (2017). The primary disease gradient of wheat stripe rust (Puccinia striiformis f. sp. tritici) across spatial scales (Ph. D. thesis), Oregon State University. Available online at: https://ir.library.oregonstate.edu/xmlui/handle/1957/59932?show=full (accessed February 1, 2020).

[B67] FRAC (2020). FRAC Code List 2020: Fungal Control Agents Sorted by Cross Resistance Pattern and Mode of Action (Including FRAC Code Numbering). Available online at: https://www.frac.info/docs/default-source/publications/frac-code-list/frac-code-list-2020-final.pdf?sfvrsn=8301499a_2 (accessed February 2020).

[B68] French R. D. (2016). Fungicides Labeled for Wheat for the Control of Rusts in Texas. Texas A&M AgriLife Extension. Available online at: http://amarillo.tamu.edu/files/2016/03/Fungicides-for-rusts-on-wheat-Spring-2016.pdf (accessed February 1, 2020).

[B69] Gangwar O. P. Bhardwaj S. C. Kumar S. Prasad P. Khan H. Savadi S. (2017). “Overcoming stripe rust of wheat: a threat to food security,” in Management of Wheat and Barley Diseases, ed D. P. Singh (Palm Bay, FL: Apple Academic Press, 115–131.

[B70] Gaunt R. E. Cole M. J. (1991). An analysis of yield reduction caused by stripe rust in Rongotea wheat. Aust. J. Agric. Res. 42, 45–52. 10.1071/AR9910045

[B71] Germán S. Azzimonti G. Castro M. García R. Quincke M. Pereyra S. (2018). Roya estriada de trigo: epidemia en 2017 asociada a la presencia de razas agresivas del patógeno y sus posibles consecuencias. Rev. INIA 54, 36–41.

[B72] Gisi U. Sierotzki H. Cook A. McCaffery A. (2002). Mechanisms influencing the evolution of resistance to Q_o_ inhibitor fungicides. Pest Manag. Sci. 58, 859–867. 10.1002/ps.565 12233175

[B73] Grabow B. S. Shah D. A. DeWolf E. D. (2016). Environmental conditions associated with stripe rust in Kansas winter wheat. Plant Dis. 100, 2306–2312. 10.1094/PDIS-11-15-1321-RE 30682905

[B74] Gupta I. L. Sharma B. S. Dalela G. G. (1975). Fungicidal control of yellow rust of barley. Indian J. Mycol. Plant Pathol. 5:115.

[B75] Han Q. M. Kang Z. S. Buchenauer H. Huang L. L. Zhao J. (2006). Cytological and immunocytochemical studies on the effects of the fungicide tebuconazole on the interaction of wheat with stripe rust. J. Plant Pathol. 88, 263–271. 10.4454/jpp.v88i3.871

[B76] Han Q. M. Kang Z. S. Wei G. R. Wang X. Z. (2003). Studies on folicur and caramba for the control of wheat stripe rust disease. Plant Protec. 29, 61–63.

[B77] Hardison J. R. (1963). Commercial control of *Puccinia striiformis* and other rusts in seed crops of *Poa pratensis* by nickel fungicides. Phytopathology 53, 209–216.

[B78] Hardison J. R. (1966). Systemic activity of two derivatives of 1,4-oxathiin against smut and rust diseases of grasses. Plant Dis. Report. 50:624.

[B79] Hardison J. R. (1975a). Control of *Puccinia striiformis* by two new systemic fungicides, Bay MEB 6447 and BAS 31702 F. Plant Dis. Rep. 59, 652–655.

[B80] Hardison J. R. (1975b). Relationships of molecular structure of 1,4-oxathiin fungicides to chemotherapeutic activity against rust and smut fungi in grasses. Phytopathology 61, 731–735. 10.1094/Phyto-61-731

[B81] He C. Zhang Y. Zhou W. Guo Q. Bai B. Shen S.. (2019). Study on stripe rust (*Puccinia striiformis*) effect on grain filling and seed morphology building of special winter wheat germplasm Huixianhong. PLoS ONE 14:e0215066. 10.1371/journal.pone.0215066 31112545 PMC6528950

[B82] Hendrix J. W. (1994). Stripe rust, what it is and what to do about it. Wash. State Univ. Stn. Circ. 424, 1–6.

[B83] Hewitt H. G. (1998). Fungicides in Crop Protection. Wallingford: CAB International.

[B84] Hims M. J. Cook R. J. (1991). The use of rainfall and accumulated mean temperature to indicate fungicide activity in the control of leaf diseases of winter wheat in the UK. Bull. OEPP/EPPO Bull. 21, 477–484.

[B85] Hollaway G. (2019). Stripe Rust of Wheat. Agriculture Victoria. Available online at: http://agriculture.vic.gov.au/__data/assets/pdf_file/0008/402767/Stripe-rust-of-wheat-AG1167-March-2019.pdf (accessed February 1, 2020).

[B86] Hollomon D. W. (2015). “Fungicide resistance: 40 years on and still a major problem,”. in Fungicide Resistance in Plant Pathogens. Principles and a Guide to Practical Management, eds H. Ishii and D. W. Hollomon (Tokyo: Springer, 3–11.

[B87] Hovmøller M. S. Justesen A. F. Brown J. K. M. (2002). Clonality and long-distance migration of *Puccinia striiformis* f. sp. *tritici* in north-west Europe. Plant Path. 51, 24–32. 10.1046/j.1365-3059.2002.00652.x

[B88] Hovmøller M. S. Sørensen C. K. Walter S. Justesen A. F. (2011). Diversity of *Puccinia striiformis* on cereals and grasses. Annu. Rev. Phytopathol. 49, 197–217. 10.1146/annurev-phyto-072910-095230 21599494

[B89] Hovmøller M. S. Walter S. Bayles R. A. Hubbard A. Flath K. Sommerfeldt N. (2016). Replacement of the European wheat yellow rust population by new races from the centre of diversity in the near-Himalayan region. Plant Pathol. 65, 402–411. 10.1111/ppa.12433

[B90] Hovmøller M. S. Yahyaoui A. H. Milus E. A. Justesen A. F. (2008). Rapid global spread of two aggressive strains of a wheat rust fungus. Mol. Ecol. 17, 3818–3826. 10.1111/j.1365-294X.2008.03886.x 18673440

[B91] Ivic D. (2010). “Curative and eradicative effects of fungicides,” in Fungicides, ed C. Odile (InTech). 10.5772/13766 Available online at: https://www.intechopen.com/books/fungicides/curative-and-eradicative-effects-of-fungicides (accessed February 1, 2020).

[B92] Jackson-Ziems T. A. Giesler L. J. Harveson R. M. Wegulo S. N. Korus K. Adesemoye A. O. (2016). “Fungicide application timing and disease control,” in Papers in Plant Pathology, 486. Available online at: https://digitalcommons.unl.edu/cgi/viewcontent.cgi?article=1487&context=plantpathpapers (accessed February 1, 2020).

[B93] Jin Y. (2011). Role of *Berberis* spp. as alternate hosts in generating new races of *Puccinia graminis* and *P. striiformis*. Euphytica 179, 105–108. 10.1007/s10681-010-0328-3

[B94] Jin Y. Szabo L. J. Carson M. (2010). Century-old mystery of *Puccinia striiformis* life history solved with the identification of *Berberis* as an alternate host. Phytopathology 100, 432–435. 10.1094/PHYTO-100-5-0432 20373963

[B95] Jørgensen L. Nielsen B. (1994). Control of yellow rust (*Puccinia striiformis*) on winter wheat by ergosterol inhibitors at full and reduced dosages. Crop Prot. 13, 323–330. 10.1016/0261-2194(94)90045-0

[B96] Jørgensen L. N. Oliver R. P. Heick T. M (2018). “Occurrence and avoidance of fungicide resistance in cereal diseases,” in Integrated Disease Management of Wheat and Barley, ed R. Oliver (Cambridge: Burleigh Dodds Science Publishing), 235–259. 10.19103/AS.2018.0039.13

[B97] Jørgensen N. L. Hovmøller M. S. Hansen J. G. Lassen P. Clarkc B. (2014). IPM strategies and their dilemmas including an introduction to www.eurowheat.org. J. Integr. Agric. 13, 265–281. 10.1016/S2095-3119(13)60646-2

[B98] Kang Z. Zhao J. Han D. Zhang H. Wang X. Wang C.. (2010). “Status of wheat rust research and control in China,” in Proceedings of BGRI, Technical Workshop, 30–31 May 2010 (St Petersburg). Available online at: https://globalrust.org/content/status-wheat-rust-research-and-control-china (accessed February 1, 2020).

[B99] Kang Z. H. Li X. Wan A. M. Wang M. N. Chen X. M. (2019). Differential sensitivity among *Puccinia striiformis* f. sp. *tritici* isolates to propiconazole and pyraclostrobin fungicides. Can. J. Plant Pathol. 41, 415–434. 10.1080/07060661.2019.1577301

[B100] Kiran K. Rawal H. Dubey H. Jaswal R. Bhardwaj S. C. Prasad P.. (2017). Dissection of genomic features and variations of three pathotypes of *Puccinia striiformis* through whole genome sequencing. Sci. Rep. 7:42419. 10.1038/srep42419 28211474 PMC5314344

[B101] Klymiuk V. Yaniv E. Huang L. Raats D. Fatiukha A. Chen S.. (2018). Cloning of the wheat Yr15 resistance gene sheds light on the plant tandem kinase-pseudokinase family. Nat. Commun. 9:3735. 10.1038/s41467-018-06138-9 30282993 PMC6170490

[B102] Lan C. Randhawa M. S. Huerta-Espino J. Singh R. P. (2017). “Genetic analysis of resistance to wheat rusts,” in Methods in Molecular Biology, ed S. Periyannan (New York, NY: Humana Press), 137–149. 10.1007/978-1-4939-7249-4_12 28856647

[B103] Line R. F. (1993). Integrated pest management for wheat: IPM in a wide-ranging system. Plant Dis. 77, 303–307.

[B104] Line R. F. (2002). Stripe rust of wheat and barley in North America: a retrospective historical review. Annu. Rev. Phytopathol. 40, 75–118. 10.1146/annurev.phyto.40.020102.111645 12147755

[B105] Line R. F. Chen X. M. (1995). Successes in breeding for and managing durable resistance to wheat rusts. Plant Dis. 79, 1254–1255. 10.1094/PD-79-1254

[B106] Line R. F. Qayoum A. (1992). Virulence, aggressiveness, evolution and distribution of races of *Puccinia striiformis* (the cause of stripe rust of wheat) in North America, 1968–87. *USDA Bull* 1788:44.

[B107] Liu H. W. Xia Z. T. Chang Y. L. (1965). Field experiments on the application of sulfanilic acid for controlling stripe rust of wheat in Shensi province. Chin. J. Plant Protec. 4, 341–345.

[B108] Liu L. Wang M. Feng J. See D. R. Chen X. (2019). Whole-genome mapping of stripe rust resistance quantitative trait loci and race specificity related to resistance reduction in winter wheat cultivar Eltan. Phytopathol. 109, 1226–1235. 10.1094/PHYTO-10-18-0385-R 30730788

[B109] Liu T. Wan A. Liu D. Chen X. (2017). Changes of races and virulence genes in *Puccinia striiformis* f. sp. *tritici*, the wheat stripe rust pathogen, in the United States from 1968 to 2009. Plant Dis. 101, 1522–1532. 10.1094/PDIS-12-16-1786-RE 30678601

[B110] Loladze A. Druml T. Wellings C. R. (2009). “Differential adaptation of Australian and New Zealand stripe rust isolates to high temperature,” in 12th International Cereal Rusts and Powdery Mildews Conference, 13–15 October (Antalya), 22.

[B111] Lucas J. A. Hawkins N. J. Fraaije B. A. (2015). The evolution of fungicide resistance. Adv. Appl. Microbiol. 90, 29–92. 10.1016/bs.aambs.2014.09.001 25596029

[B112] Ma L. Qiao J. Kong X. Zou Y. Xu X. Chen X.. (2015). Effect of low temperature and wheat winter-hardiness on survival of *Puccinia striiformis* f. sp. *tritici* under controlled conditions. PLoS ONE 10:e0130691. 10.1371/journal.pone.0130691 26083371 PMC4470655

[B113] Maccaferri M. Zhang J. Bulli P. Abate Z. Chao S. Cantu D.. (2015). A genome-wide association study of resistance to stripe rust (*Puccinia striiformis* f. sp. *tritici*) in a worldwide collection of hexaploid spring wheat (*Triticum aestivum* L.). G3-Genes Genom. Genet. 5, 449–465. 10.1534/g3.114.014563 25609748 PMC4349098

[B114] Marchal C. Zhang J. Zhang P. Fenwick P. Steuernagel B. Adamski N. M.. (2018). BED-domain-containing immune receptors confer diverse resistance spectra to yellow rust. Nat. Plants 4, 662–668. 10.1038/s41477-018-0236-4 30150615

[B115] Mboup M. Bahri B. Leconte M. De Vallavieille-Pope C. Kaltz O. Enjalbert J. (2012). Genetic structure and local adaptation of European wheat yellow rust populations: the role of temperature-specific adaptation. Evol. Appl. 5, 341–352. 10.1111/j.1752-4571.2011.00228.x 25568055 PMC3353355

[B116] McIntosh R. Mu J. Han D. Kang Z. (2018). Wheat stripe rust resistance gene Yr24/Yr26: A retrospective review. Crop J. 6, 321–329. 10.1016/j.cj.2018.02.001

[B117] McLean M. Henry F. Hollaway G. (2010). “Stripe rust management in wheat.,” in BCG 2010 Season Research Results, 140–142. Available online at: https://www.farmtrials.com.au/trial/14049 (accessed February 1, 2020).

[B118] Milus E. A. Kristensen K. Hovmøller M. (2009). Evidence for increased aggressiveness in a recent widespread strain of *Puccinia striiformis* f. sp. *tritici* causing stripe rust of wheat. Phytopathology 99, 89–94. 10.1094/PHYTO-99-1-0089 19055439

[B119] Mu J. Huang S. Liu S. Zeng Q. Dai M. Wang Q.. (2019). Genetic architecture of wheat stripe rust resistance revealed by combining QTL mapping using SNP-based genetic maps and bulked segregant analysis. Theor. Appl. Genet. 132, 443–455. 10.1007/s00122-018-3231-2 30446795

[B120] Muleta K. T. Rouse M. N. Rynearson S. Chen X. Buta B. G. Pumphrey M. O. (2017). Characterization of molecular diversity and genome-wide mapping of loci associated with resistance to stripe rust and stem rust in Ethiopian bread wheat accessions. BMC Plant Biol. 17:134. 10.1186/s12870-017-1082-7 28778144 PMC5545024

[B121] Murray G. (2004). Stripe Rust–Spray Thresholds, Economics of Control and Risks From Sucker Varieties. GRDC Research Updates. Available online at: https://archive.lls.nsw.gov.au/__data/assets/pdf_file/0003/495300/archive-fungicidal-control-of-stripe-rust.pdf (accessed February 1, 2020).

[B122] Murray G. Wellings C. Simpfendorfer S. Cole C. (2005). Stripe Rust: Understanding the Disease in Wheat. Department of Primary Industries, State of New South Wales. Available online at: https://www.dpi.nsw.gov.au/__data/assets/pdf_file/0006/158964/stripe-rust-in-wheat.pdf (accessed February 1, 2020).

[B123] Murray G. M. Brennan J. (2009). Estimating disease losses to the Australian wheat industry. Australas. Plant Pathol. 38, 558–570. 10.1071/AP09053

[B124] Murray G. M. Ellison P. J. Watson A. (1995). Effects of stripe rust on the wheat plant. Australas. Plant Pathol. 24, 261–270. 10.1071/APP9950261

[B125] Murray G. M. Ellison P. J. Watson A. Cullis B. R. (1994). The relationship between wheat yield and stripe rust as affected by length of epidemic and temperature at the grain development stage of crop growth. Plant Pathol. 43, 397–405. 10.1111/j.1365-3059.1994.tb02701.x

[B126] Nagarajan S. Singh D. V. (1990). Long-distance dispersion of rust pathogens. Annu. Rev. Phytopathol. 28, 139–153. 10.1146/annurev.py.28.090190.001035 20540608

[B127] Napier B. A. S. Bayles R. A. Stigwood P. L. (2000). “Sensitivity of powdery mildew and yellow rust to DMI, morpholine and strobilurin fungicides in England and Scotland,” in The BCPC Conference: Pests and Diseases, Volume 1. Proceedings of an International Conference Held at the Brighton Hilton Metropole Hotel, 13–16 November 2000 (Brighton, 427–434.

[B128] Naruoka Y. Garland-Campbell K. A. Carter A. H. (2015). Genome-wide association mapping for stripe rust (*Puccinia striiformis* f. sp. *tritici*) in US Pacific Northwest winter wheat (*Triticum aestivum* L.). Theor. Appl. Genet. 128, 1083–1101. 10.1007/s00122-015-2492-2 25754424

[B129] NCERA (2019). Fungicide Efficacy for Control of Wheat Diseases. North Central Regional Committee on Management of Small Grain Diseases. Available online at: https://www.ag.ndsu.edu/extplantpath/publications-newsletters/crop-disease-control/NCERA184Wheatfungicidetable_2019.pdf (accessed February 1, 2020).

[B130] Nsabiyera V. Bariana H. S. Qureshi N. Wong D. Hayden M. J. Bansal U. K. (2018). Characterisation and mapping of adult plant stripe rust resistance in wheat accession Aus27284. Theor. Appl. Genet. 131, 1459–1467. 10.1007/s00122-018-3090-x 29560515

[B131] Oliver R. P. (2014). A reassessment of the risk of rust fungi developing resistance to fungicides. Pest. Manag. Sci. 70, 1641–1645. 10.1002/ps.3767 24616024

[B132] Peng F. Miu S. Yang Z. Fu Y. Yang Y. Yu Y. (2020). Rapid quantification of fungicide effectiveness on inhibiting wheat stripe rust pathogen (*Puccinia striiformis* f. sp. *tritici*). Plant Dis. 10.1094/PDIS-09-19-1836-RE 32649268

[B133] Poole N. F. Arnaudin M. E. (2014). The role of fungicides for effective disease management in cereal crops. Can. J. Plant Pathol. 36, 1–11. 10.1080/07060661.2013.870230

[B134] Powelson R. L. Shaner G. E. (1966). An effective chemical seed treatment for systemic control of seedling infection of wheat by stripe rust (*Puccinia striiformis*). Dis. Report. 50, 806–807.

[B135] Price C. L. Parker J. E. Warrilow A. G. Kelly D. E. Kelly S. L. (2015). Azole fungicides–understanding resistance mechanisms in agricultural fungal pathogens. Pest Manag. Sci. 71, 1054–1058. 10.1002/ps.4029 25914201

[B136] Purdy L. H. (1964). Inhibition of wheat stripe rust by the antibiotic phleomycin in the greenhouse. Plant Dis. Rep. 48, 159–161.

[B137] Rahmatov M. Otambekova M. Muminjanov H. Rouse M. N. Hovmøller M. S. Nazari K. (2019). Characterization of stem, stripe and leaf rust resistance in Tajik bread wheat accessions. Euphytica 215:55 10.1007/s10681-019-2377-6

[B138] Rakotondradona R. Line R. F. (1984). Control of stripe rust and leaf rust of wheat with seed treatments and effects of treatments on the host. Plant Dis. 68, 112–117. 10.1094/PD-68-112

[B139] Ramachandran S. R. Mueth N. A. Zheng P. Hulbert S. H. (2020). Analysis of miRNAs in two wheat cultivars infected with *Puccinia striiformis* f. sp. *tritici*. Front. Plant Sci. 10:1574. 10.3389/fpls.2019.01574 31998329 PMC6965360

[B140] Rapilly F. (1979). Yellow rust epidemiology. Annu. Rev. Phytopathol. 17, 59–73. 10.1146/annurev.py.17.090179.000423

[B141] Rathmell W. G. Skidmore A. M. (1982). Recent advances in the chemical control of cereal rust diseases. Outlook Agr. 11, 37–43.

[B142] Reid D. Swart J. (2004). Evaluation of Foliar Fungicides for the Control of Stripe Rust (Puccinia striiformis) in SRWW in the Northern Texas Blacklands. Available online at: http://agrilife.org/amarillo/files/2010/11/evaluationof_foliar_fungicides_2004.pdf (accessed February 1, 2020).

[B143] Reis E. M. Carmona M. A. (2011). “Sensibilidade de razas de *Puccinia triticina* a fungicidas,” in II Simposio Nacional De Agricultura, FAGRO–GTI Agricultura y IPNI Cono Sur. 29 y 30 de setiembre de 2011, Paysandu (Montevideo: Universidad de la Repúbica), 89–94.

[B144] Reis E. M. Carmona M. A. (2013). “Classification of fungicides,” in Fungicides: Classification, Role in Disease Management and Toxicity Effects, eds M. N. Wheeler and B. R. Johnston (New York, NY: Nova Science Publishers, 91–104.

[B145] Ren R. S. Wang M. N. Chen X. M. Zhang Z. J. (2012). Characterization and molecular mapping of Yr52 for high-temperature adult-plant resistance to stripe rust in spring wheat germplasm PI 183527. Theor. Appl. Genet. 125, 847–857. 10.1007/s00122-012-1877-8 22562146

[B146] Rosewarne G. M. Singh R. P. Huerta-Espino J. Herrera-Foessel S. A. Forrest K. L. Hayden M. J.. (2012). Analysis of leaf and stripe rust severities reveals pathotype changes and multiple minor QTLs associated with resistance in an Avocet × Pastor wheat population. Theor. Appl. Genet. 124:1283. 10.1007/s00122-012-1786-x 22274764

[B147] Saleem K. Sørensen C. K. Labouriau R. Hovmøller M. S. (2019). Spatiotemporal changes in fungal growth and host responses of six yellow rust resistant near-isogenic lines of wheat. Plant Pathol. 68, 1320–1330. 10.1111/ppa.13052

[B148] SARDI (2020). Cereal Seed Treatments 2020. Summary of 2019 Season and Implications for 2020. South Australian Research and Development Institute. Available online at: https://pir.sa.gov.au/__data/assets/pdf_file/0005/237920/Cereal_seed_treatments_2020.pdf (accessed February 1, 2020).

[B149] Schmitz H. K. Medeiros C. A. Craig I. R. Stammler G. (2014). Sensitivity of *Phakopsora pachyrhizi* towards quinone-outside-inhibitors and demethylation-inhibitors, and corresponding resistance mechanisms. Pest Manag. Sci. 70, 378–388. 10.1002/ps.3562 23589453

[B150] Schwessinger B. (2017). Fundamental wheat stripe rust research in the 21st century. New Phytol. 213, 1625–1631. 10.1111/nph.14159 27575735

[B151] Schwessinger B. Chen Y. J. Tien R. Vogt J. K. Sperschneider J.. (2019). Distinct life histories impact dikaryotic genome evolution in the rust fungus *Puccinia striiformis* causing stripe rust in wheat. bioRxiv 859728. 10.1101/859728 32271913 PMC7250506

[B152] Schwessinger B. Sperschneider J. Cuddy W. S. Garnica D. P. Miller M. E. Taylor J. M.. (2018). A near-complete haplotype-phased genome of the dikaryotic wheat stripe rust fungus *Puccinia striiformis* f. sp. *tritici* reveals high interhaplotype diversity. mBio 9:e02275-17. 10.1128/mBio.02275-17 29463659 PMC5821087

[B153] Scott R. B. Line R. F. (1985). Control of stripe rust and leaf rust of wheat with triadimenol seed treatments and triadimefon foliar sprays. Phytopathol. 75, 1383–1384.

[B154] Shaner G. E. Powelson R. L. (1971). Epidemiology of stripe rust of wheat, 1961–1968. OR Agric. Exp. Stn. Tech. Bull. 117:31.

[B155] Sharma R. Nazari K. Amanov A. Ziyaev Z. Jalilov A. (2016). Reduction of winter wheat yield losses caused by stripe rust through fungicide management. J. Phytopathol. 164, 671–677. 10.1111/jph.12490

[B156] Sharma-Poudyal D. Chen X. M. Wan A. M. Zhan G. M. Kang Z. S. Cao S. Q.. (2013). Virulence characterization of international collections of the wheat stripe rust pathogen, *Puccinia striiformis* f. sp. *tritici*. Plant Dis. 97, 379–386. 10.1094/PDIS-01-12-0078-RE 30722363

[B157] Singh R. Mahmoudpour A. Rajkumar M. Narayana R. (2017a). A review on stripe rust of wheat, its spread, identification and management at field level. Res. Crops 18, 528–533. 10.5958/2348-7542.2017.00091

[B158] Singh R. Srivastava P. Sharma A. Bains N. S (2017b). Bread wheat cultivar PBW 343 carries residual additive resistance against virulent stripe rust pathotype. J. Crop Improv. 31, 183–191. 10.1080/15427528.2016.1263262

[B159] Singh V. K, Mathuria, R. C. Gogoi R. Aggarwal R. (2016). Impact of different fungicides and bioagents, and fungicidal spray timing on wheat stripe rust development and grain yield. Indian Phytopath. 69, 357–362.

[B160] Siyoum G. Z. Zeng Q. D. Zhao J. Chen X. M. Badebo A. Tian Y.. (2019). Inheritance of virulence and linkages of virulence genes in an Ethiopian isolate of the wheat stripe rust pathogen (*Puccinia striiformis* f. sp. *tritici*) determined through sexual recombination on *Berberis holstii*. Plant Dis. 103, 2451–2459. 10.1094/PDIS-02-19-0269-RE 31322491

[B161] Solh M. Nazari K. Tadesse W. Wellings C. R. (2012). “The growing threat of stripe rust worldwide,” in BGRI 2012 Technical Workshop, 1–4 September 2012 (Beijing).

[B162] Tang C. Wei J. Han Q. Liu R. Duan X. Fu Y.. (2015). PsANT, the adenine nucleotide translocase of *Puccinia striiformis*, promotes cell death and fungal growth. Sci. Rep. 5:11241. 10.1038/srep11241 26058921 PMC4462048

[B163] Tian Y. Meng Y. Zhao X. Chen X. Ma H. Xu S.. (2019). Trade-off between triadimefon sensitivity and pathogenicity in a selfed sexual population of *Puccinia striiformis* f. sp. *tritici*. Front. Microbiol. 10:2729. 10.3389/fmicb.2019.02729 31849881 PMC6901989

[B164] Tian Y. Zhan G. M. Chen X. M. Tungruentragoon A. Lu X. Zhao J.. (2016). Virulence and simple sequence repeat marker segregation in a *Puccinia striiformis* f. sp. *tritici* population produced by selfing a Chinese isolate on *Berberis shensiana*. Phytopathology 106, 185–191. 10.1094/PHYTO-07-15-0162-R 26551448

[B165] Turner S. Stougaard B. Bohannon B. (2016). Stripe Rust Fungicide Trials for Winter and Spring Wheat at NWARC. Montana State University. Available online at: http://agresearch.montana.edu/nwarc/presentations/documents/04rust.pdf (accessed February 1, 2020).

[B166] Vergara-Diaz O. Kefauvera S. C. Elazaba A. Nieto-Taladrizb M. T. Araus J. L. (2015). Grain yield losses in yellow-rusted durum wheat estimated using digital and conventional parameters under field conditions. Crop J. 3, 200–210. 10.1016/j.cj.2015.03.003

[B167] Viljanen-Rollinson S. L. H. Marroni M. V. Butler R. C. (2010). Benefits from plant resistance in reducing reliance on fungicides in cereal disease management. N. Z. Plant Prot. 63, 145–150. 10.30843/nzpp.2010.63.6556

[B168] Viljanen-Rollinson S. L. H. Parkes R. A. Armour T. Cromey M. G. (2002). Fungicide control of stripe rust in wheat: protection or eradication. N. Z. Plant Prot. 55, 336–340. 10.30843/nzpp.2002.55.3902

[B169] Wallwork H. Garrard T. (2020). Cereal Seed Treatments 2020. Summary of 2019 Season and Implications for 2020. Available online at: https://pir.sa.gov.au/__data/assets/pdf_file/0005/237920/Cereal_seed_treatments_2020.pdf (accessed February 1, 2020).

[B170] Wamalwa M. N. Owuoche J. Ogendo J. Wanyera R. (2019). Multi-pathotype testing of selected kenyan wheat germplasm and watkin landraces for resistance to wheat stripe rust (*Puccinia striiformis* f. sp *tritici*) Races. Agronomy 9:770 10.3390/agronomy9110770

[B171] Wan A. Wang X. Kang Z Chen. X. (2017). “Variability of the stripe rust pathogen,” in Stripe Rust, eds X. Chen and Z. Kang (Dordrecht: Springer), 35–154. 10.1007/978-94-024-1111-9

[B172] Wan A. Zhao Z. Chen X. He Z. Jin S. Jia Q.. (2004). Wheat stripe rust epidemic and virulence of *Puccinia striiformis* f. sp. *tritici* in China in 2002. Plant Dis. 88, 896–904. 10.1094/PDIS.2004.88.8.896 30812521

[B173] Wan A. M. Chen X. M. He Z. H. (2007). Wheat stripe rust in China. Aust J Agric Res. 58, 605–619. 10.1071/AR06142

[B174] Wan A. M. Chen X. M. Yuen J. (2016). Races of *Puccinia striiformis* f. sp. *tritici* in the United States in 2011 and 2012 and comparison with races in 2010. Plant Dis. 100, 966–975. 10.1094/PDIS-10-15-1122-RE 30686156

[B175] Wang B. Sun Y. Song N. Zhao M. Liu R. Feng H.. (2017). *Puccinia striiformis* f. sp. *tritici* microRNA-like RNA 1 (Pst-milR1), an important pathogenicity factor of *Pst*, impairs wheat resistance to *Pst* by suppressing the wheat pathogenesis-related 2 gene. New Phytol. 215, 338–350. 10.1111/nph.14577 28464281

[B176] Wang J. Tao F. Tian W. Guo Z. Chen X. Xu X.. (2017). The wheat WRKY transcription factors TaWRKY49 and TaWRKY62 confer differential high-temperature seedling-plant resistance to *Puccinia striiformis* f. sp. *tritici*. PLoS ONE 12:e0181963. 10.1371/journal.pone.0181963 28742872 PMC5526533

[B177] Wang Z. Zhao J. Chen X. Peng Y. Ji J. Zhao S.. (2016). Virulence variations of *Puccinia striiformis* f. sp. *tritici* isolates collected from *Berberis* spp. in China. Plant Dis. 100, 131–138. 10.1094/PDIS-12-14-1296-RE 30688563

[B178] Wanyera R. Macharia J. K. Kilonzo S. (2010). Challenges of Fungicide Control on Wheat Rusts in Kenya. IntechOpen. Available online at: https://www.intechopen.com/books/fungicides/challenges-of-fungicide-control-on-wheat-rusts-in-kenya (accessed February 1, 2020).

[B179] Wanyera R. Wamalwa M. Odemba M. Wanga H. Kinyanjui P. Onyango V. (2016). Management of wheat rusts at different growth stages using Nativo 300 SC (trifloxystrobin 100g/L+tebuconazole 200g/L) fungicide. Australas. J. Plant Sci. 10, 1273–1280. 10.21475/ajcs.2016.10.09.p7676

[B180] Wegulo S. (2015). Stripe Rust Confirmed in Nebraska; Scout Wheat Fields for Early Disease Detection. CROPWATCH. Available online at: https://cropwatch.unl.edu/wheat-stripe-rust (accessed February 2020).

[B181] Wellings C. R. (2011). Global status of stripe rust: a review of historical and current threats. Euphytica 179, 129–141. 10.1007/s10681-011-0360-y

[B182] Wellings C. R. McIntosh R. A. Walker J. (1987). *Puccinia striiformis* f. sp. *tritici* in eastern Australia-possible means of entry and implications for plant quarantine. Plant Pathol. 36, 239–241. 10.1111/j.1365-3059.1987.tb02230.x

[B183] WSU (2018). Fungicide Data. Washington State University. Available online at: https://striperust.wsu.edu/disease-management/fungicide-data/ (accessed February 2020).

[B184] WSU (2019). Fungicide Data. Washington State University. Available online at: https://striperust.wsu.edu/disease-management/fungicide-data/ (accessed February 1, 2020).

[B185] Xi K. Kumar K. Holtz M. D. Turkington T. K. Chapman B. (2015). Understanding the development and management of stripe rust in central Alberta. Can. J. Plant Pathol. 37, 21–39. 10.1080/07060661.2014.981215

[B186] Xia C. Wang M. Yin C. (2018). Genomic insights into host adaptation between the wheat stripe rust pathogen (*Puccinia striiformis* f. sp. *tritici*) and the barley stripe rust pathogen (*Puccinia striiformis* f. sp. *hordei*). *BMC Genomics* 19:664 10.1186/s12864-018-5041-y PMC6134786 30208837

[B187] Xu Q. Tang C. Wang L. Zhao C. Kang Z. Wang X. (2020). Haustoria–arsenals during the interaction between wheat and *Puccinia striiformis* f. sp. *tritici*. Mol. Plant Pathol. 21, 83–94. 10.1111/mpp.12882 31774224 PMC6913192

[B188] Xu Q. Tang C. Wang X.. (2019). An effector protein of the wheat stripe rust fungus targets chloroplasts and suppresses chloroplast function. Nat. Commun. 10:5571. 10.1038/s41467-019-13487-6 31804478 PMC6895047

[B189] Yang M. Li G. Wan H. Li L. Li J. Yang W.. (2019). Identification of QTLs for stripe rust resistance in a recombinant inbred line population. Int. J. Mol. Sci. 20:3410. 10.3390/ijms20143410 31336736 PMC6678735

[B190] Yang Y. Yu Y. Bi C. (2016). Quantitative proteomics reveals the defense response of wheat against *Puccinia striiformis* f. sp. *tritici*. Sci. Rep. 6:34261 10.1038/srep34261 27678307 PMC5039691

[B191] Young C. S. Paveley N. D. Vaughan T. B. Thomas J. M. Lockley K. D. (2003). Predicting epidemics of yellow rust (*Puccinia striiformis*) on the upper canopy of wheat from disease observations on lower leaves. Plant Pathol. 52, 338–349. 10.1046/j.1365-3059.2003.00848.x

[B192] Yuan C. Y. Wang M. N. Skinner D. Z. See D. R. Xia C. J. Guo X. H.. (2018). Inheritance of virulence, construction of a linkage map, and mapping of virulence genes in *Puccinia striiformis* f. sp. *tritici* by virulence and molecular characterization of a sexual population through genotyping-by-sequencing. Phytopathology 108, 133–141. 10.1094/PHYTO-04-17-0139-R 28876207

[B193] Yuan F. P. Zeng Q. D. Wu J. H. Wang Q. L. Yang Z. J. Liang B. P.. (2018). QTL mapping and validation of adult plant resistance to stripe rust in chinese wheat landrace Humai 15. Front. Plant Sci. 9:968. 10.3389/fpls.2018.00968 30026752 PMC6041984

[B194] Zadoks J. C. Chang T. T. Konzak C. F. (1974). A decimal code for the growth stages of cereals. Weed Res. 14, 415–421.

[B195] Zegeye H. Rasheed A. Makdis F. Badebo A. Ogbonnaya F. C. (2014). Genome-wide association mapping for seedling and adult plant resistance to stripe rust in synthetic hexaploid wheat. PLoS ONE 9:e105593. 10.1371/journal.pone.0105593 25153126 PMC4143293

[B196] Zeng Q. Wu J. Liu S.. (2019). A major QTL co-localized on chromosome 6BL and its epistatic interaction for enhanced wheat stripe rust resistance. Theor. Appl. Genet. 132, 1409–1424. 10.1007/s00122-019-03288-2 30707240

[B197] Zeng Q. D. Han D. J. Wang Q. L. (2014). Stripe rust resistance and genes in Chinese wheat cultivars and breeding lines. Euphytica 196, 271–284. 10.1007/s10681-013-1030-z

[B198] Zeng S. M. Luo Y. (2006). Long-distance spread and interregional epidemics of wheat stripe rust in China. Plant Dis. 90, 980–988. 10.1094/PD-90-0980 30781287

[B199] Zhang C. Huang L. Zhang H. Hao Q. Lyu B. Wang M.. (2019). An ancestral NB-LRR with duplicated 3′UTRs confers stripe rust resistance in wheat and barley. Nat. Commun. 10:4023. 10.1038/s41467-019-11872-9 31492844 PMC6731223

[B200] Zhang H. Y. Wang Z. Ren J. D. Du Z. Y. Quan W. Zhang Y. B.. (2017). A QTL with major effect on reducing stripe rust severity detected from a Chinese wheat landrace. Plant Dis. 101, 1533–1539. 10.1094/PDIS-08-16-1131-RE 30678599

[B201] Zhao J. Wang L. Wang Z. Chen X. Zhang H. Yao J.. (2013). Identification of eighteen berberis species as alternate hosts of *Puccinia striiformis* f. sp. *tritici* and virulence variation in the pathogen isolates from natural infection of barberry plants in China. Phytopathology 103, 927–934. 10.1094/PHYTO-09-12-0249-R 23514262

[B202] Zhao J. Wang M. N. Chen X. M. Kang Z. S. (2016). Role of alternate hosts in epidemiology and pathogen variation of cereal rusts. Annu. Rev. Phytopathol. 54, 207–228. 10.1146/annurev-phyto-080615-095851 27296143

[B203] Zheng W. Huang L. Huang J. Wang X. Chen X. Zhao J.. (2013). High genome heterozygosity and endemic genetic recombination in the wheat stripe rust fungus. Nat. Commun. 4:2673. 10.1038/ncomms3673 24150273 PMC3826619

